# Fast, precise and cloning-free knock-in of reporter sequences *in vivo* with high efficiency

**DOI:** 10.1242/dev.201323

**Published:** 2023-06-29

**Authors:** Yiran Zhang, Katy Marshall-Phelps, Rafael Góis de Almeida

**Affiliations:** Centre for Discovery Brain Sciences, University of Edinburgh, Edinburgh, EH16 4SB, UK

**Keywords:** HDR, Homology-directed repair, Knock-in, Neuron, Synaptobrevin, Vamp, Zebrafish

## Abstract

Targeted knock-in of fluorescent reporters enables powerful gene and protein analyses in a physiological context. However, precise integration of long sequences remains challenging *in vivo*. Here, we demonstrate cloning-free and precise reporter knock-in into zebrafish genes, using PCR-generated templates for homology-directed repair with short homology arms (PCR tagging). Our novel knock-in reporter lines of vesicle-associated membrane protein (vamp) zebrafish homologues reveal subcellular complexity in this protein family. Our approach enables fast and efficient reporter integration in the zebrafish genome (in 10-40% of injected embryos) and rapid generation of stable germline-transmitting lines.

## Teaser

**Summary:** Optimization of CRISPR-Cas9 assisted knock-in in zebrafish using PCR generated HDR templates, exemplified in vamp/synaptobrevin genes

## INTRODUCTION

Zebrafish (*Danio rerio*) are well suited for genetic manipulations (Driever et al., 1996; Haffter et al., 1996; Howe et al., 2013) and live-imaging of molecules, cells and tissues *in vivo* over time. Transgenesis, e.g. *tol2*-based, is widely used to express fluorescent protein (FP) reporters in zebrafish (Kwan et al., 2007), but transgenes integrate randomly and multiple times in the genome (Kawakami, 2007). Tol2 transgenes also typically contain limited regulatory promoter/enhancer sequences which, coupled with positional effects of random integration (Mosimann et al., 2013; Roberts et al., 2014), may not accurately replicate endogenous expression patterns (Bussmann and Schulte-Merker, 2011). Furthermore, when transgenic strategies are used to express a protein of interest fused to an FP to study its role *in vivo*, transgenes are expressed simultaneously with the endogenous protein, and the ensuing non-physiological protein concentrations limit the validity of localization or functional analyses (Stadler et al., 2013). In addition, cloning long promoter or coding sequences to assemble transgenes can be challenging.

CRISPR-based (Hwang et al., 2013; Jao et al., 2013) genome editing has revolutionized studies of gene function and cellular processes *in vivo*. Cas9 protein, guided to a target locus by a guide RNA (gRNA), catalyses a double-stranded break (DSB) (Cong et al., 2013; Jinek et al., 2012). This triggers DNA repair, e.g. by the error-prone non-homologous end-joining (NHEJ) pathway, or instead by homology-directed repair (HDR), a competing pathway that is engaged only when a DNA template homologous to the DSB region is present (Ceccaldi et al., 2016). HDR templates can be exploited to create genetic modifications, such as inserting reporter sequences in frame with endogenous genes. This type of reporter ‘knock-in’ ensures that the reporter is expressed under the control of the full endogenous regulatory sequence of the gene of interest, and of similar chromatin accessibility. Knock-ins enable powerful analyses of endogenous proteins in their physiological context (Schwinn et al., 2020), circumventing overexpression-associated artifacts. However, the low efficiency of HDR compared with NHEJ (Sonoda et al., 2006), and the laborious cloning of long homology arms (>0.5 kbp) previously considered necessary for HDR, lead to limited implementation, gauged by the scarcity of reports of in-frame zebrafish knock-in reporter lines (Hisano et al., 2015; Hoshijima et al., 2016; Shin et al., 2014; Wierson et al., 2020). Efficient zebrafish reporter knock-in would facilitate optical access to native cellular processes *in vivo*.

**Fig. 1.**
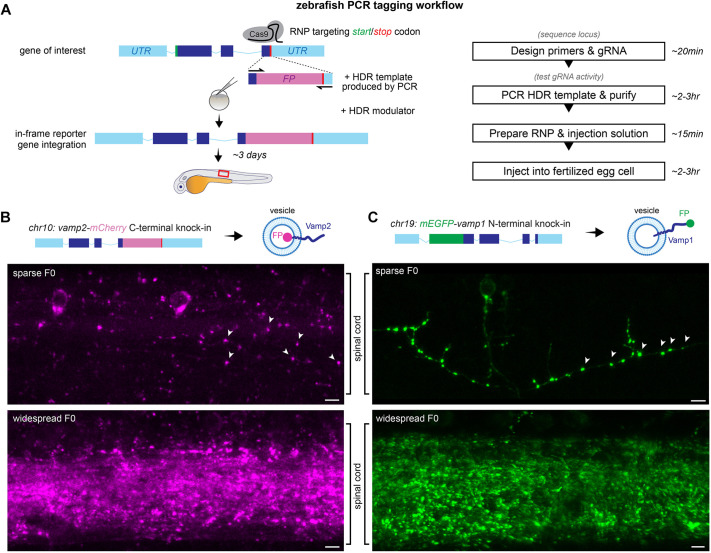
**PCR tagging in zebrafish.** (A) PCR tagging workflow: a gRNA/Cas9 RNP targeting the start or stop codon of a gene of interest is co-injected with a PCR-generated HDR template with short homology arms into fertilized zebrafish eggs, leading to integration of a fluorescent protein (FP) coding sequence. UTR, untranslated region. Red box indicates field of view in B,C and throughout the figures. (B,C) Lateral view of spinal cords of 3-5 dpf *vamp2-mCherry* and *mEGFP-vamp1* PCR-tagged zebrafish showing vesicular fluorescence in synaptic boutons (arrowheads). Scale bars: 5 µm.

To establish an efficient, cloning-free and precise reporter knock-in strategy, we sought to combine several knock-in optimization strategies recently described *in vivo* and *in vitro* (Gutierrez-Triana et al., 2018; Yu et al., 2020). We reasoned that the first step of introducing a DSB can be efficiently achieved with a ribonucleoprotein (RNP) formed by Cas9 protein and synthetic CRISPR gRNAs, which have been shown to be highly active in zebrafish (Hoshijima et al., 2019; Keatinge et al., 2021; Kroll et al., 2021) and obviate the need for cloning and/or *in vitro* transcription. For cloning-free production of the HDR template, we adapted ‘PCR tagging’ (Fueller et al., 2020), which uses linear dsDNA templates quickly synthesized by high-fidelity PCR, as fluorescent reporter sequences are longer (>700 nt) than commercially-available ssDNA synthesis typically allows. Recent *in vivo* studies, including in zebrafish, have shown that short homology arms in the 30-100 bp range are sufficient to drive HDR in different gene-editing strategies (Kanca et al., 2019; Liu et al., 2022; Seleit et al., 2021; Wierson et al., 2020). These short arms can be included in the oligonucleotide primers and incorporated in the template during PCR. Furthermore, recent studies have sought to tip the NHEJ-HDR balance following Cas9 cleavage to favour HDR via small-molecule HDR activators or NHEJ repressors, which appear to increase HDR in zebrafish (Aksoy et al., 2019; Murakami and Kobayashi, 2022). We therefore sought to incorporate HDR enhancers in the workflow, by adding them directly to the solution injected into fertilized eggs. We reasoned that even if knock-in rates are low, zebrafish egg production rate and microinjection throughput are not limiting (e.g. a single pair mating can lay hundreds of fertilized eggs in a morning). Furthermore, using fluorescence as readout of knock-in success should enable rapid screening of many embryos to identify successfully tagged animals for further analysis and to screen for germline transmission, which would preclude alternative laborious genetic analyses of all injected embryos e.g. by PCR. Given the predicted fast turnaround from design to fluorescent readout, we reasoned that this approach, if successful, would greatly facilitate knock-ins in the zebrafish model (Fig. 1A).

## RESULTS

### Design of PCR tagging strategy for zebrafish vamp genes

To test PCR tagging in zebrafish, we sought to integrate fluorescent reporter sequences in frame with vesicle-associated membrane protein (vamp) genes, which are essential for neuronal development and function. Vamp genes encode transmembrane SNARE (soluble N-ethylmaleimide sensitive factor attachment protein receptor) proteins that drive fusion of cellular membranes (Urbina and Gupton, 2020). For example, Vamp2 is the main homologue present on synaptic vesicles throughout the nervous system, and is required for vesicle fusion at synaptic terminals to drive neurotransmission (Archer et al., 1990). Different Vamp homologues drive membrane fusion in different organelles or cell types, but their complex expression patterns partially overlap and the extent to which they functionally substitute for each other *in vivo* is unclear (Bhattacharya et al., 2002; Parlati et al., 2000). The need to carefully dissect Vamp expression and localization *in vivo* is underscored by the complexity of disease phenotypes caused by mutations in human vamp genes (Verhage and Sørensen, 2020). Importantly, Vamp protein subcellular localization and trafficking depend on their expression levels (Gordon et al., 2016). Therefore, it would be preferable to avoid reporter overexpression approaches for Vamp protein analyses. Knock-in reporters of endogenous Vamp proteins would thus facilitate further studies of their individual roles *in vivo*, both in health and disease.

To test whether PCR tagging would be functional in distinct loci, coding termini and using various fluorescent reporters, we first targeted pan-neuronally expressed *vamp2* on chromosome 10 and *vamp1*, a related homologue located on chromosome 19 expressed in specific neuronal subpopulations such as motor neurons (Liu et al., 2011) and inhibitory interneurons (Vuong et al., 2018). We first formed gRNA/Cas9 RNP targeting the *vamp2* stop codon or the *vamp1* start codon and verified that they were highly active in zebrafish embryos using a restriction digestion assay (Fig. S1A-D). We then combined the RNPs with PCR-generated HDR templates. The *vamp2-mCherry* HDR template encoded a short five amino acid linker and mCherry coding sequences (720 bp), flanked by 30-60 bp of *vamp2* sequence preceding the stop codon on the left homology arm, and with the stop codon and downstream untranslated region in the right homology arm. The HDR template omitted the mCherry start codon to prevent expression from out-of-frame integration and altered the gRNA target sequence to prevent repeated cleavage (Fig. S1E). The *mEGFP-vamp1* HDR template encoded the monomeric EGFP coding sequence followed by a short linker (729 bp), flanked by 30-60 bp of *vamp1* sequence preceding and containing the *vamp1* start codon in the left homology arm, and 30-60 bp of the *vamp1* coding sequence downstream of its start codon (Fig. S1F).

As Vamp1/2 proteins are mostly vesicular, correct integration should lead to punctate mCherry or mEGFP expression in the zebrafish nervous system. We injected RNP and HDR template into single-cell zebrafish eggs and raised the embryos until 3-4 days post-fertilization (dpf), when we then sought to detect mCherry or mEGFP signal in the central nervous system. We readily detected punctate mCherry or mEGFP fluorescent signal in the brain and spinal cord of injected (F0) embryos, ranging from sparse labelling to widespread expression (representative views of the spinal cord in Fig. 1B,C). In more sparsely labelled larvae, mCherry and mEGFP could readily be detected at boutons along axonal projections, likely synaptic terminals (e.g. arrowheads in Fig. 1B,C), suggesting that integration occurred in the predicted *vamp1/2* locus, and that Vamp1/2-tagged endogenous protein could be produced and localized as expected. To confirm that fluorescent signal in F0 larvae represented correct integration in the target genomic loci, we performed PCR on the 5′ and 3′ knock-in junctions in injected larvae, using a *vamp1/2* gene-specific primer combined with an mCherry- or mEGFP-specific primer (Fig. S2A,D). Although no product could be amplified from control wild-type genomic DNA or from F0 larvae injected only with RNP, we could amplify the expected junctions from F0 larvae injected with both RNP and HDR template. In particular, we readily obtained PCR products of the approximately correct size from F0 larvae that exhibited fluorescent signal, sparse or widespread, although also occasionally from larvae that did not exhibit fluorescence, suggesting that in those embryos knock-in occurred mosaically and in cells that do not normally express Vamp1/2, or occurred imprecisely, disrupting the reporter reading frame (Fig. S2B,E). We sequenced PCR products obtained from F0 larvae, which confirmed the presence of the expected sequence as well as of imprecise knock-in in some instances (Fig. S2C,F). Thus, the PCR tagging approach is applicable for knocking-in fluorescent reporters in target zebrafish genomic loci.

### Determining initial parameters for efficient PCR tagging in zebrafish

**Fig. 2.**
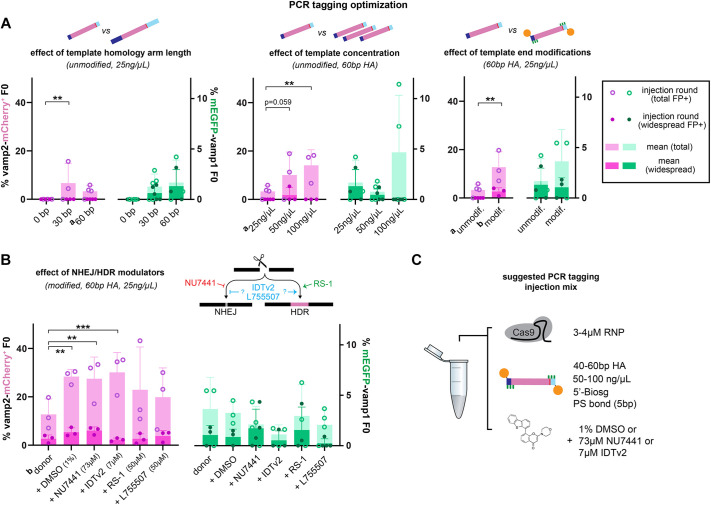
**Optimization of PCR tagging injection mix.** (A,B) Optimization of homology arm (HA) length, template concentration and end modification (A) and small molecule modulation of NHEJ/HDR pathways (B). Efficiency is expressed as percentage of F0 larvae that are mCherry^+^ or mEGFP^+^ at 3-5 dpf. Circles indicate individual injection rounds and bars indicate the mean of 3-5 injection rounds per condition. The number of surviving larvae analyzed per injection round varied between 9 and 253 (88 on average, for full details see Table S2). Error bars indicate standard deviation. *P<0.05, **P<0.01, ***P<0.001 (two-way ANOVA corrected for multiple comparisons). For ease of comparison, data indicated by superscript a and b are shown in more than one graph. (C) Suggested components of injection mix for PCR tagging in zebrafish. Biosg, standard biotin modification; PS, phosphorothioate bond.

To establish efficient PCR tagging in F0 larvae, we tested the effect of varying components of the PCR tagging injection mix on the proportion of embryos that showed sparse, widespread or no fluorescence at 3-4 dpf (Fig. 2A,B). We first tested the effect of the length of the homology arms on the HDR template on knock-in efficiency. We noted that adding any template to the injection mix was enough to decrease the survival and normal morphological development of injected larvae at 3-4 dpf from the >95% of RNP-only injections to 50-70%. This indicates that the simple presence of dsDNA HDR template in the injection mix causes some developmental toxicity (Fig. S3). Compared with control injections with a template without homology arms, injecting templates with either 30 bp- or 60 bp-long homology arms promoted knock-in, such that 1-7% of surviving larva at 3-4 dpf showed mEGFP or mCherry fluorescence in the nervous system (Fig. 2A). We then tested increasing template concentration in the injection mix. This was the only parameter that caused a significant dose-dependent decrease in survival, such that 100 ng/µl of template reduced survival to <25% (Fig. S3). Nevertheless, increasing template concentration from 25 ng/µl to 100 ng/µl tripled the knock-in efficiency (to 5-14%, Fig. 2A). We then tested adding chemical end-modifications to the HDR template, which have been shown to improve knock-in precision or efficiency in other systems. Adding a biotin moiety has been shown to reduce undesirable end-to-end multimeric arrangements of template molecules in target loci; and adding phosphorothioate bonds has been shown to protect the template from degradation by exonuclease activity (Gutierrez-Triana et al., 2018; Yu et al., 2020). These modifications can easily be included when ordering oligonucleotide primers to generate the template by PCR and therefore do not delay the workflow. We found that injections with the end-modified template led to the same survival rates as unmodified template (Fig. S3), and significantly increased tagging efficiency 2- to 3-fold compared with unmodified template with the same homology arm length at the same concentration (Fig. 2A). Furthermore, modified templates increased the efficiency of recovering precisely knocked-in larvae (see below).

We then also sought to test the effect of including small-molecule modulators of NHEJ/HDR pathways in the PCR tagging injection mix (Fig. 2B). Recently, the DNA-PK inhibitor NU7441, the RAD51-activator RS-1 and the β3-adrenergic receptor agonist L755507 were shown to increase HDR in zebrafish, medaka or other systems (Aksoy et al., 2019; Murakami and Kobayashi, 2022). We tested adding these HDR modulators, the proprietary Alt-R™ IDT HDR Enhancer V2 from Integrated DNA Technologies or a DMSO-only vehicle control to the PCR tagging injection mix. Inclusion of HDR modulators at the concentrations used (73 µM NU7441, 50 µM RS-1, 50 µM L755507, 7 µM IDT HDR Enhancer V2 – all in 1% DMSO) did not affect survival compared to injections without modulators at 3-4 dpf (Fig. S3). Interestingly, all conditions, including the DMSO vehicle, increased the proportion of fluorescent *vamp2-mCherry* tagged larvae compared with not including any small molecule in the mix, between 1.6- and 2.4-fold. This increase reached statistical significance for DMSO, NU7441 and IDT HDR enhancer V2. This enhancement did not occur in *mEGFP-vamp1*-tagged larvae, indicating that factors that limit the efficiency of PCR tagging are locus-specific. Indeed, throughout all conditions tested, we observed statistically significant differences when changing parameters of *vamp2-mCherry* tagging only. Nevertheless, *mEGFP-vamp1* tagging efficiency also increased on average with higher template concentration and template end-modification, even if not reaching the same significance threshold. We note that *mEGFP-vamp1* tagging is generally less efficient than *vamp2-mCherry* tagging. This is unlikely to be because of lower activity of the *vamp1*-targeting RNP, which appears to perform better than the *vamp2* RNP in a restriction digest assay (Fig. S1C,D). This suggests that other factors remain to be explored that affect the efficiency of knock-in in zebrafish, and that they may be locus specific. Indeed, our experiments tested a limited initial selection of parameters, and it will be important to further optimize PCR tagging. For example, in preliminary experiments (Fig. S4), we increased RNP concentration, tested combinations of NHEJ/HDR modulators and tested an improved primer/template design that included truncated *vamp2* stop codon gRNA-target sequences. The RNP is capable of binding to, but not cleaving, such short <16 bp sequences, and this may co-transport the template to the nucleus and increase the concentration available there for HDR (Feng et al., 2021). Indeed, these preliminary experiments yielded the highest proportion of Vamp2-mCherry^+^ F0 larvae obtained in a single injection round, 49% (25/51; Fig. S4A), indicating that further optimization is possible. Such improvements do not alter the workflow, which remains cloning-free.

Given our data, we suggest the following conditions for the PCR tagging injection mix in zebrafish: 50-100 ng/μl of HDR template bearing 40-60 bp homology arms and 5′-biotin and phosphorothioate bond modifications, and adding 1% DMSO, 73 µM NU7441 or 7 µM IDT HDR enhancer v2 (Fig. 2C).

### PCR tagging is applicable to additional zebrafish genomic loci

**Fig. 3.**
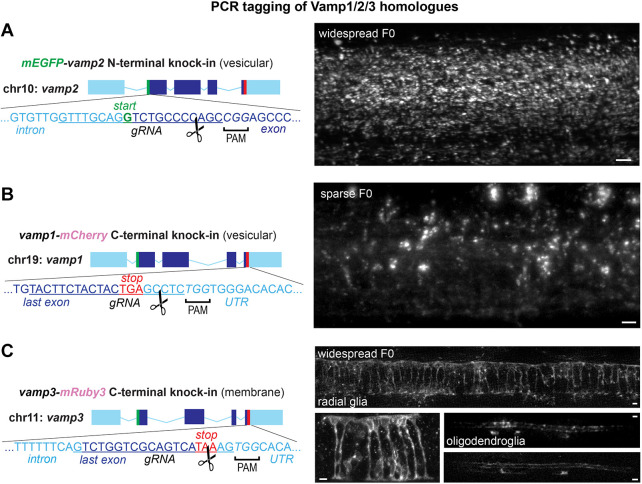
**PCR tagging of related homologues Vamp1, Vamp2 and Vamp3.** (A-C). Targeting additional *vamp2* (N-terminal, A), *vamp1* (C-terminal, B) and *vamp3* (C-terminal, C) genomic loci. Representative widespread and sparse F0 larvae show vesicular mEGFP-Vamp2 and Vamp1-mCherry throughout the spinal cord and a membrane-like expression pattern of Vamp3-mRuby3 in radial and myelinating glial cells in the spinal cord. Scissors indicate predicted RNP cleavage site. Scale bars: 5 µm.

We then sought to test whether the PCR tagging approach was also efficient in other genomic loci. 40-50 bp homology arms and 50-70 ng/µl template also enabled N-terminal *mEGFP-vamp2* tagging, C-terminal *vamp1-mCherry* and *vamp1-mRuby3* tagging and C-terminal tagging of *vamp3-mRuby3* (Fig. 3A-C; Fig. S4B). *vamp3* is a vamp homologue located on chromosome 11 that is expressed in glia and other non-neuronal cells (McMahon et al., 1993). Unlike PCR-tagged Vamp1/2 protein expression, which is mostly vesicular, Vamp3-mRuby3 expression appeared to be located on the membrane of the expressing cells, highlighting their morphology, in agreement with the described Vamp3 protein localization throughout the endomembrane system. We observed cells with an oligodendrocyte-like morphology and radial glia-like morphology throughout the spinal cord of Vamp3-mRuby3^+^ F0 larvae (Fig. 3C). These results suggest that the PCR tagging approach is broadly applicable to both N and C termini in various loci of the zebrafish genome. Given the straightforward workflow, tagging of both termini can be performed in parallel for genes of interest in which the optimal target is not known *a priori*. Given the speed of this cloning-free approach, high efficiency of labelling, coupled with the non-limiting production of zebrafish eggs and high-throughput of the injection procedure, we expect that PCR tagging will be a useful addition to the zebrafish genetic manipulation toolkit.

### N-terminal tagging enables assessment of the precision of integration

**Fig. 4.**
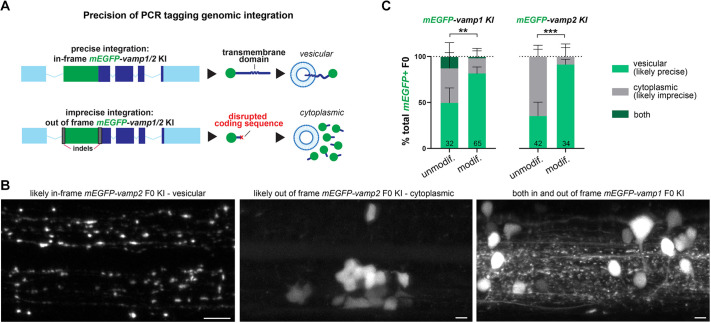
**N-terminal Vamp1/2 knock-in reveals precision level of PCR tagging.** (A,B) N-terminal tagging allows detection of imprecise integration e.g. when indels knock the *vamp1/2* coding sequences out of frame, disrupting vesicular localization signals and leading to cytoplasmic FP expression. Examples of F0 larva with likely precise (vesicular) and imprecise (cytoplasmic) mEGFP knock-in into *vamp1/2* shown in B. (C) The proportion of *vamp1/2* PCR-tagged larvae with likely precise (vesicular) expression pattern is higher when using end-modified HDR templates versus unmodified templates. Number of 3-4 dpf mEGFP^+^ F0 larva indicated in bars (includes experiments in Fig. 2 and Fig. S4). Error bars indicate 95% CI calculated using the Wilson/Brown method. **P<0.01 or ***P<0.001 (Fisher's test for vesicular versus cytoplasmic/mixed expression). Scale bars: 5 µm.

N-terminal tagging of Vamp1/2 proteins enabled us to assess the precision of PCR tagging-mediated reporter sequence integration. The vesicular membrane localization of Vamp1/2 proteins is determined by targeting signals in and around the transmembrane domain (Grote et al., 1995). If PCR tagging does not knock-in the reporter sequence precisely in frame, for example due to non-HDR-mediated template integration, erroneous multimeric arrangements of the linear dsDNA template molecules, or the presence of accompanying indels flanking the reporter sequence, then translation of the endogenous Vamp1/2 vesicle targeting signals would be disrupted and the vesicular localization of the N-terminal mEGFP compromised (Fig. 4A). Indeed, with both *mEGFP-vamp1* and *mEGFP-vamp2* tagging, we observed examples of potentially imprecise *mEGFP* integration, whereby F0 larvae had cytoplasmic rather than vesicular mEGFP fluorescence in nervous system cells (Fig. 4B). We also observed individual larvae with both vesicular (likely precise) and cytoplasmic (likely imprecise) mEGFP localization, which suggests the occurrence of multiple knock-in events in the rapidly dividing embryonic cells after injections into the fertilized egg. We then reasoned that at least some of the imprecise integration events could be due to multimeric arrangements or concatenation of the HDR template, an occurrence which the end-modification of the template with bulky moieties such as biotin should reduce. To test this, we compared the proportions of vesicular, cytoplasmic or mixed mEGFP-expressing F0 larvae in our N-terminal *vamp1/2* tagging experiments using unmodified and modified HDR templates. We found that larvae injected with modified templates exhibited vesicular mEGFP more frequently than cytoplasmic mEGFP expression, compared with unmodified templates, likely representing higher frequencies of precise reporter sequence integration (Fig. 4C). Together with the enhancement in F0 larvae tagging efficiency they provide (Fig. 2A), this supports the use of modified templates to generate novel knock-in alleles.

### Germline transmission of PCR tagged zebrafish genes

We then raised FP^+^ F0 larvae to adulthood to identify founders with germline transmission of novel *vamp1/2/3*-tagged alleles (Table S1). Of six Vamp2-mCherry*^+^* F0 adults that were injected with end-modified templates, three (50%) were founders (two had widespread mCherry expression and one had sparse expression). F1 transmission rates were 1-36% (Table S1). PCR analyses and Sanger sequencing of junctions confirmed precise integration (Fig. S5A-D). In contrast, from 17 Vamp2-mCherry^+^ F0 adults that were injected with unmodified templates, one (6%) transmitted a *vamp2-mCherry* allele to 4% of its F1 offspring. PCR amplification of F1 genomic sequences at the 5′ and 3′ integration junctions showed a correct amplicon around the 5′ junction, but a product longer than expected around the 3′ junction (Fig. S5B). Sanger sequencing revealed a second partial mCherry coding sequence copy following the first (Fig. S5A,E). This further suggests that end-modified templates achieve more precise integration, including in the germline. We also screened 12 Vamp2-mCherry adults without mCherry fluorescence (injected with unmodified templates) and none transmitted a *vamp2-mCherry* allele to its F1 offspring; we have also been unable to isolate N-terminal tagged mEGFP-vamp2 founders from the six mEGFP^+^ F0 adults screened so far (Table S1).

We also identified two *vamp1-mRuby3* founders from four F0 animals with widespread mRuby3 expression (transmitting the knocked-in allele to 4-20% of their F1 offspring), and two *vamp3-mRuby3* founders from three F0 animals with widespread mRuby3 expression (transmitting the knocked-in allele to 20-50% of their F1 offspring). PCR analyses (Fig. S5F) and Sanger sequencing of junctions confirmed precise integration. Three and seven F0 adults with sparse mRuby3 expression were not founders for *vamp1* and *vamp3* homologues, respectively.

Collectively, 6/9 F0 animals (67%) with widespread expression of a fluorescent reporter tagged with end-modified template at the C terminus of three vamp homologues were founders of novel, precisely knocked-in alleles. In comparison, 1/14 F0 animals (7%) with sparse expression was a founder. This is consistent with previous reports of a correlation between somatic editing and germline transmission in zebrafish HDR (Aksoy et al., 2019). Thus, the ability to pre-screen F0 larvae for fluorescent reporters as a readout of PCR-tagging integration will greatly aid the success rate of isolating novel knock-in alleles that are transmitted to the F1 generation. All cells in F1 animals and the subsequent offspring should be edited, and indeed larvae no longer exhibited mosaic reporter expression in *vamp*1/2/3-expressing cells (see examples in Movies 1-4). Our novel *vamp1-mRuby3*, *vamp2-mCherry* and *vamp3-mRuby3* lines will enable future comparative analyses of endogenous Vamp1/2/3 proteins and Vamp1/2/3-bearing vesicles and organelles *in vivo*, without incurring reporter overexpression artefacts.

### Versatility of PCR tagging design enables cellular analyses of gene expression

**Fig. 5.**
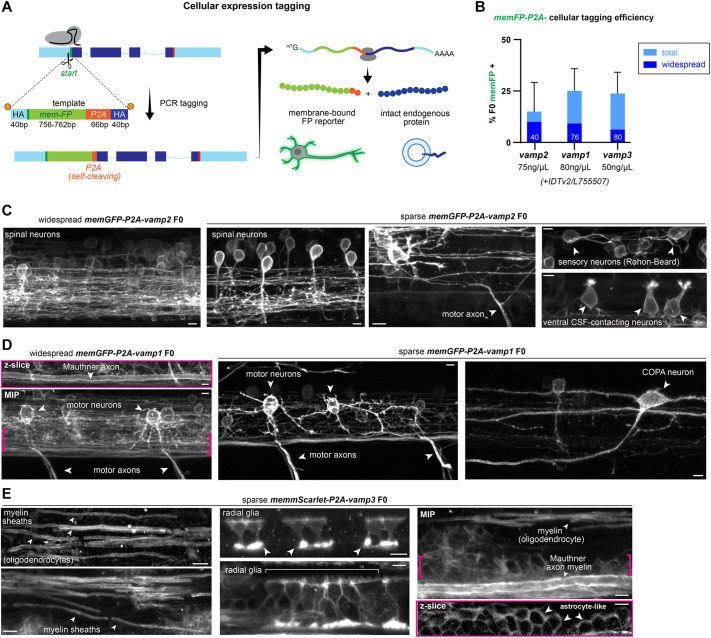
**Using PCR tagging for cellular expression analysis reveals vamp cellular diversity.** (A) Cellular expression tagging strategy in which a membrane-bound fluorescent protein sequence (memFP) followed by a ‘self-cleaving’ P2A peptide is knocked into the N terminus of a gene of interest. During mRNA translation, ribosomal skipping leads to separate production of memFP outlining the morphology of the expressing cells and of intact endogenous protein. HA, homology arms; m7G/AAAA, mRNA 5′-cap and polyadenylation signal. (B) Efficiency of cellular expression tagging of *vamp1/2/3* homologues. Bars represent one injection round; the number of 3-5 dpf larvae analyzed *n* is indicated within bars. Error bars indicate 95% confidence intervals for FP^+^ expression (Wilson/Brown calculation). Modified HDR template concentration indicated below bars; 30 µM IDT HDR enhancer v2 or 80 µM L755507 was added in *vamp1/2* or in *vamp3* tagging injections, respectively. (C) *vamp2* cellular expression tagging shows extensive neuronal expression throughout the spinal cord, e.g. in sensory Rohon–Beard neurons, CSF-contacting neurons and motor neurons. (D) *vamp1* cellular expression tagging reveals expression in motor neurons, premotor reticulospinal neurons (e.g. the Mauthner cell) and interneuron subtypes such as commissural primary ascending (COPA) neurons. Brackets indicate region of the single *z*-slice shown above. (E) *vamp3* cellular expression tagging reveals primarily glial expression e.g. in myelinating oligodendrocytes, radial glia and astrocyte-like cells in the ventral spinal cord. Brackets indicate region of *z*-slice shown below. Arrowheads point to examples of the labelled cells or structures in each panel. MIP, maximum intensity projection of *z*-stack. Scale bars: 5 µm.

We then reasoned that the high efficiency of knock-in facilitated PCR tagging could also be used for different types of genetic modification. To exemplify the versatility of PCR tagging, we developed a ‘cellular expression tag’ by knocking-in a membrane-bound reporter sequence followed by the short P2A peptide (Kim et al., 2011; Liu et al., 2017) at the start codon of target genes. The 22-amino acid long P2A peptide from porcine teschovirus-1 induces ribosomal skipping during translation leading to separate production of the membrane-bound reporter outlining the morphology of the target gene-expressing cells, and of the untagged endogenous target protein. The size of the HDR template for this approach remains under 1000 bp (Fig. 5A).

Injecting 50-80 ng/µl of end-modified HDR templates bearing 40 bp homology arms was sufficient to label 15-25% of F0 larvae with membrane reporters in *vamp1/2/3*-expressing cells (Fig. 5B). As expected for the described pan-neuronal *vamp2* expression pattern, multiple neuronal subtypes and their axonal and dendritic projections were brightly labelled in the spinal cord, including motor neurons (identified by their axons exiting the spinal cord in ventral locations); mechanosensory neurons, such as the glutamatergic Rohon–Beard cells (identified by their stereotypical dorsal location, large soma and axonal projections); and spinal interneurons, such as GABAergic cerebrospinal fluid-contacting sensory neurons (Fig. 5C). Cellular expression tagging experiments of *vamp1* also labelled neurons, more prominently motor neurons and premotor reticulospinal neurons (such as the Mauthner cell, identified by its very large diameter axon in a stereotypical ventral location), and suggest that specific spinal subpopulations such as commissural primary ascending interneurons (COPA, identified by stereotypical T-shaped dendritic projections and commissural contralateral ascending axon) also express *vamp1* (Fig. 5D, see also COPA neuron labelled by cytoplasmic mEGFP in Fig. 4B). In contrast, cellular expression tagging of *vamp3* readily labelled multiple glial cell types, such as oligodendrocytes (identified by the myelin sheaths they wrap around axonal segments), radial glia-like cells that extend their membrane from the central canal to the pial surface, and cells located in the ventral spinal cord that resemble bona-fide mammalian astrocytes recently described by Chen and colleagues (Chen et al., 2020) (Fig. 5E).

Together with the direct protein-tagging experiments, these results indicate that in zebrafish, as in mammals, neurons primarily express *vamp1/2*, whereas glia primarily express *vamp3*. Our results also suggest that specific neuronal subpopulations, such as motor neurons, co-express the highly homologous *vamp1* and *vamp2* genes *in vivo*. Furthermore, these data indicate that cellular expression tagging of endogenous genes could be used as a live-imaging alternative to complement other techniques to study gene expression, such as *in situ* hybridization approaches.

### Simultaneous PCR tagging of two vamp homologues

**Fig. 6.**
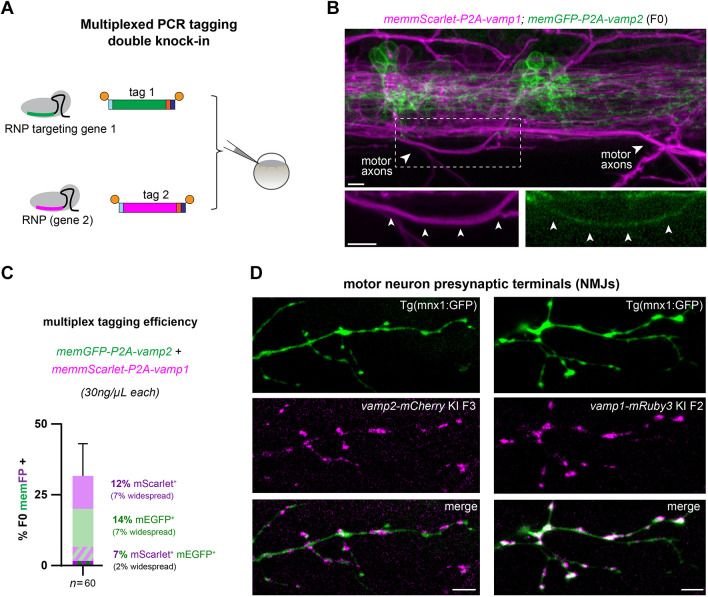
**Multiplexed cellular expression tagging reveals *vamp1/2* co-expression in motor axons.** (A) Our optimized PCR tagging conditions facilitate multiplex knock-in in two target loci simultaneously. (B) Example memmScarlet-2A-*vamp1*^+^; memEGFP-2A-*vamp2*^+^ F0 larva shows partially overlapping expression patterns, e.g. in motor axons (arrowheads). (C) Efficiency of double cellular tagging of *vamp1*/*2*, shown as percentage of F0 larvae showing memEGFP and/or memmScarlet fluorescence. Bars represent one injection round, error bars indicate 95% confidence intervals for FP^+^ expression (Wilson/Brown calculation). (D) *vamp1-mRuby3* or *vamp2-mCherry* knock-in lines crossed with transgenic motor neuron reporter mnx1:GFP confirms that both Vamp1 and Vamp2 proteins are found at the presynaptic terminals of motor neurons, i.e. the neuromuscular junctions that innervate the trunk musculature. Scale bars: 5 µm.

Given the high efficiency of the PCR tagging approach, we then tested whether multiplexed knock-in of two genes simultaneously could be achieved with a single injection (Fig. 6A). We sought to target *vamp1* and *vamp2* homologues in parallel, with two spectrally separate reporters in the same individual animals, given that our cellular expression tagging experiments suggested co-expression of both homologues in motor neurons. Double knock-in of *memEGFP-2A-vamp2*; *memmScarlet-2A-vamp1* using 30 ng/µl of each template led to simultaneous memEGFP and memmScarlet expression in 4/60 (7%) F0 larvae, in addition to single memEGFP^+^ or memmScarlet^+^ larvae (Fig. 6B,C). Spinal cord imaging of double knock-in larvae supported our observation of *vamp1* and *vamp2* co-expression in individual motor neurons with single-gene PCR tagging, as we readily identified memmScarlet^+^ memEGFP^+^ motor axons by their stereotyped trajectory exiting the ventral spinal cord (Fig. 6B).

The functional significance of *vamp1/2* co-expression in specific neuronal subtypes, such as motor neurons, is poorly understood *in vivo* (Liu et al., 2019). It is also unclear whether, within individual neurons, Vamp1 and Vamp2 protein localization and roles are redundant. We therefore sought to validate that both proteins had a presynaptic localization, by imaging motor axon terminals at the musculature using an established transgenic motor neuron reporter line, Tg(mnx1:GFP), crossed with our novel *vamp2-mCherry* and *vamp1-mRuby* knock-in lines. We confirmed that both Vamp2 and Vamp1 proteins localized at presynaptic terminals along individual motor axons at the neuromuscular junctions, identified as GFP^+^ boutons (Fig. 6D). This suggests that both proteins perform a similar function at synapses.

### PCR tagging reveals heterogeneity masked by reporter overexpression strategy

**Fig. 7.**
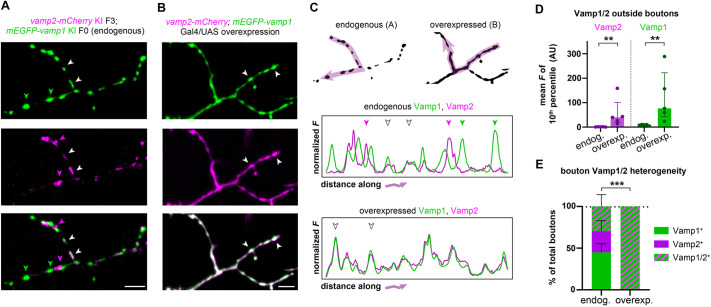
**Multiplexed PCR tagging reveals *vamp1/2* co-expression in motor axons.** (A) A larva in which endogenous Vamp1/2 proteins are both tagged shows that synaptic boutons in individual motor axons exhibit molecular heterogeneity and can be Vamp1^+^ (green arrowheads), Vamp2^+^ (magenta arrowheads) or both Vamp1/2^+^ (white arrowheads). (B) An individual motor axon in which Vamp1/2 proteins are labelled using a Gal4/UAS overexpression strategy. All synaptic boutons appear to be Vamp1/2^+^ (white arrowheads). (C) mEGFP and mCherry fluorescence intensity profiles along the indicated arrows in motor axon terminals shown in A,B. Arrowheads indicate the terminals highlighted in A,B. Note mEGFP and mCherry overlap in overexpressed but not endogenously labelled terminals. (D) To estimate the fluorescence signal along the axon outside the synaptic boutons, the lowest 10% of the fluorescence intensity value was averaged per axon profile (plotted in arbitrary units, AU). Note significant increase in overexpression strategy. Circles indicate individual axon profiles (five axons from three endogenously tagged larvae and five axons from five overexpressing larvae) and bars indicate the median. Error bars indicate the interquartile range. **P<0.01 (two-tailed Mann–Whitney test). (E) The proportion of Vamp1^+^, Vamp2^+^ and Vamp1/2^+^ boutons along each motor axon. The overexpression strategy masks neuromuscular junction molecular heterogeneity. Bars indicate the proportion of each type of bouton averaged from five axons each (from three endogenously tagged and five overexpressing larvae), and error bars indicate the 95% confidence intervals (Wilson/Brown calculation). ***P<0.0001 (Fisher's exact test). Scale bars: 5 µm.

To further explore the specificity of Vamp1/2 protein localization, we then knocked-in *mEGFP-vamp1* into our *vamp2-mCherry* line, and imaged motor axon terminals. Interestingly, we observed that some boutons were mEGFP^+^ only, others were mCherry^+^ only, and yet others were doubly EGFP^+^ mCherry^+^ (Fig. 7A), revealing heterogeneity in Vamp1/2 composition of presynaptic terminals along motor axons. We then sought to compare our approach of tagging endogenous Vamp1/2 genes with the more common approach of reporter overexpression used for protein localization studies. To perform an equivalent experiment using the Gal4/UAS overexpression system (Halpern et al., 2008), we injected a Tol2 expression construct, in which the *vamp2-mCherry* and *mEGFP-vamp1* cDNAs were under the regulatory control of UAS repeats, into fertilized eggs of the established transgenic line Tg(mnx1:Gal4). This led to co-expression of both fusion proteins in sparse motor neurons, individual axon terminals of which were then imaged using similar microscope acquisition parameters to the PCR tagging experiment. Remarkably, we observed that Vamp2-mCherry and mEGFP-Vamp1 reporter fusion proteins co-localized in all synaptic boutons imaged, and that the axonal terminal outside of the boutons exhibited an elevated mCherry and mEGFP signal (Fig. 7B). Plotting the normalized fluorescence profiles along axonal terminals highlighted the molecular heterogeneity of endogenously tagged Vamp1/2 boutons (Fig. 7C). Compared with endogenously tagged axons, overexpressing axons showed significantly increased fluorescence outside of the boutons (using the 10th percentile of the fluorescent intensity range as a proxy; Fig. 7D) and complete coincidence of mCherry and mEGFP localization at all terminals (Fig. 7E). Thus, overexpressing Vamp1/2 fusion reporters in addition to the endogenous proteins mislocalized these predominantly presynaptic proteins and completely masked the endogenous molecular heterogeneity present along individual axons.

These experiments independently validate that Vamp1 and Vamp2 proteins can be co-expressed in motor neurons and reveal that boutons along the same axon become molecularly distinct, carrying Vamp1, Vamp2 or a combination of both. Importantly, our comparison to an overexpression strategy underscores the need for strategies like PCR tagging, which maintain normal physiological concentrations of proteins of interest. In addition, our novel *vamp1/2/3* knock-in lines established in this study will enable future studies dissecting the functional complexity and cellular diversity of SNARE genes in the vertebrate nervous system.

## DISCUSSION

Large HDR-mediated insertions had been thought to require long homology arms (>500 bp) (Danner et al., 2017; Yang et al., 2020), requiring laborious cloning for assembly of knock-in templates. Our adaptation of PCR tagging and other recent studies (Kanca et al., 2019; Liu et al., 2022; Mi and Andersson, 2023; Ranawakage et al., 2020; Seleit et al., 2021; Wierson et al., 2020) indicate that short homology arms (30-60 bp) suffice for zebrafish knock-in. This enables using PCR for fast, cloning-free template production, including homology arms in primers containing 5′-end modifications that increase precise editing. In our hands, template concentration is a key parameter, with the highest percentage of FP^+^ F0 larvae (approaching 50%) obtained with ∼100 ng/µl of modified template. These concentrations reduced the proportion of surviving embryos (although not to limiting numbers, given the high fecundity of zebrafish). We also exploited observations that NHEJ/HDR modulators enhance knock-in in various systems, and found that DMSO, NU7441 or IDT HDR enhancer v2 significantly increased *vamp2-mCherry* tagging (Fig. 2). Interestingly, our data suggest that DMSO by itself may be responsible for most of the enhancement, as other small molecules did not enhance tagging further. Our data are in agreement with recent studies reporting that the same low DMSO concentration used (1%) also increased knock-in in human stem cells, potentially by modulating DNA accessibility for the Cas9 enzyme (Stratigopoulos et al., 2018; Usher et al., 2022). Combined with end-modified templates at concentrations <100 ng/µl, our PCR tagging conditions enabled high survival and knock-in rates. Our preliminary experiments with higher RNP concentrations, improved template design and combinations of small molecule enhancers (Fig. S4) indicate that there is scope for further systematic optimization, and that knock-in rate in F0 animals and potentially F1 transmission can be further improved. This could involve automated high-resolution quantitative vertebrate screening technologies (Early et al., 2018) to screen large compound libraries for the best HDR enhancers, to determine optimal dose response concentrations, to determine the effect of different solvents, and to identify the best combinations of enhancers of zebrafish knock-in.

### Versatility of PCR tagging

We readily achieved somatic N- or C-terminal tagging of vamp genes in injected F0 larvae as assessed by fluorescence and molecular analysis. We also identified C-terminal knock-in founders, indicating germline integration, but no N-terminal *mEGFP-vamp2* founders from six F0 adults screened so far (Table S1). N-terminal tags may interfere with Vamp2 function at an organismal level (*vamp2* knockout animals are not viable; Schoch et al., 2001), but more potential founders need to be screened to definitively test this possibility. As knock-in location may differentially interfere with endogenous protein function, we suggest that both N- and C-terminal tagging are attempted in parallel for uncharacterized genes. The high efficiency of PCR tagging suggests that cellular expression tagging (Fig. 5), which can be adapted for C-terminal tagging, can be used complementarily to *in situ* hybridization protocols to detect endogenous expression patterns. F0 generation results should be cautiously interpreted until on-target integration has been validated in the F1 generation to exclude the possibility of interpreting off-target tagging data. Furthermore, the ability to perform multiplex PCR tagging using two distinct FPs simultaneously (e.g. Fig. 6) may be particularly useful in the zebrafish model to study paralogue gene expression, given the genome duplication that occurred in the teleost lineage (Taylor et al., 2001).

Template design can also easily be adapted for other uses e.g. to introduce knock-in/knock-out reporters with termination signals at N termini, to generate floxed alleles or to introduce functional reporters (e.g. calcium, pH). It will be important in future studies to systematically test how template size affects the efficiency of knock-in. For example, Mi and Andersson recently used a similar PCR tagging approach to knock-in a larger sequence encoding Cre recombinase linked to a fluorescent reporter into four loci, albeit at lower efficiency levels, suggesting that larger template sizes become increasingly harder to knock-in. Their recent study further underscores the versatility of this approach for zebrafish genome editing (Mi and Andersson, 2023).

### Limitations of PCR tagging

The high rates of precise F1 transmission by PCR tagging are enabled by the use of fluorescence to pre-screen F0 larvae that display high levels of editing. Although simple in early embryonic/larval zebrafish, it becomes more difficult to perform as larvae grow into juveniles. Therefore, should the gene of interest only begin to be expressed at later stages, or should the intended knocked-in cassette include tags other than fluorescent reporters, alternative F0 screening may be needed. In such cases, PCR with endogenous gene- and tag-specific primers may be used to screen F0 larvae to raise and test for F1 transmission using high-throughput approaches (Kosuta et al., 2018; Zhang et al., 2020).

Incorrect integration of multimeric arrangements of linear dsDNA templates is a known potential outcome of exogenous sequence integration (Boel et al., 2018; Culp et al., 1991; Perucho et al., 1980) and may limit the usefulness of the generated lines. To date, we have isolated one founder using unmodified templates and seven using modified templates. In our *vamp2-mCherry* founder created with an unmodified template, a second partial copy of the mCherry sequence was integrated with the first in-frame full mCherry copy. Founders created using modified templates did not exhibit complete or partial copies of the reporter sequences, indicating that this strategy protects against template multimerization in zebrafish. However, small (e.g. 10-50 bp) partial duplications of the homology arms comprising the 3′ untranslated region can occur in PCR tagging, e.g. in medaka (Seleit et al., 2021). This possibility highlights the need for full sequence validation of knock-in junctions in F1 animals when establishing a novel line. As long as these duplications do not disrupt the coding sequence of the endogenous protein or reporter, their presence should not impede use of novel PCR-tagged alleles.

### Uncovering *vamp* diversity *in vivo*

Our data highlight the complexity of the SNARE repertoire *in vivo*, in particular the vamp gene family. Although Vamp2 protein is well studied, given its role at synapses (Salpietro et al., 2019; Schoch et al., 2001), Vamp1/3, which are 74-78% identical to Vamp2, have less well-characterized roles. Both can substitute for certain aspects of Vamp2 loss of function *in vitro*, with reduced efficiency (Borisovska et al., 2005; Zimmermann et al., 2014). Like Vamp2, Vamp1 mediates synaptic vesicle fusion, and a spontaneous recessive mutation in the lethal-wasting (lew) mouse model causes immobility and death by P15 (Nystuen et al., 2007). In humans, Vamp1 mutations have been identified in people with hereditary spastic ataxia 1 (Bourassa et al., 2012) and congenital myasthenic syndrome (Salpietro et al., 2017). Unlike Vamp2, Vamp1 is only expressed in specific neuronal classes including GABAergic hippocampal neurons (Vuong et al., 2018) and motor neurons (Peng et al., 2014). The significance of Vamp1/2 co-expression in these neuron types is not well understood, but in motor neurons Vamp1 eventually becomes the dominant form postnatally (Liu et al., 2019). Our analyses identified separate Vamp1^+^ or Vamp2^+^ boutons and double Vamp1^+^ Vamp2^+^ presynaptic boutons along motor axons in zebrafish larvae (Fig. 7), and it will be important to study how these proteins are dynamically regulated in individual neurons. Our novel knock-in lines will now enable dissecting the significance of Vamp1/2 molecular specialization in intact neuronal circuits *in vivo*, in the healthy nervous system and in disease models.

*vamp3* is expressed in multiple non-neuronal tissues, in which Vamp3 protein localizes throughout the endocytic pathway (McMahon et al., 1993). Within the brain, *vamp3* is expressed in neuroendocrine and glial cells and may mediate fusion of distinct vesicle populations compared with Vamp2, such as peptidergic release in astrocytes (Schwarz et al., 2017). Vamp3 is also expressed in mammalian oligodendrocytes (Feldmann et al., 2009; Lam et al., 2022; Marques et al., 2016; Zhang et al., 2014), where it has been implicated in the trafficking of specific myelin proteins and myelin biosynthesis (Feldmann et al., 2011; Lam et al., 2022). Our cellular expression tagging confirmed that *vamp3* is principally expressed in radial glia, astrocyte-like cells and myelinating oligodendrocytes in the zebrafish CNS, and our new knock-in line will help dissect the mechanistic bases of their roles in neuron and glia development and function.

### Endogenous tagging circumvents overexpression artefacts

Given the ease of transgenesis in zebrafish (e.g. Tol2-based), overexpression strategies to study protein localization or function became common in this system, for example by using the Gal4/UAS system. However, these approaches do not retain physiological concentrations of the target proteins, given that they are expressed in addition to the endogenous protein of interest. This is a particularly important consideration when the protein of interest is localized to confined subcellular compartments or small organelles such as synaptic vesicles, as there is a limited occupancy per organelle and there may be important stoichiometric relations to other organelle components needed for appropriate function. For example, *in vitro*, the balance between Vamp2 and synaptophysin, another synaptic vesicle protein, regulates vesicle trafficking along the axon and recycling dynamics (Gordon et al., 2016). In our hands, a typical overexpression strategy to study Vamp1/2 proteins in motor neurons not only mislocalized these proteins outside of synaptic boutons, but also masked an inter-bouton heterogeneity that was obvious in endogenously tagged experiments. Therefore, studies employing transgenic Vamp-reporter fusion proteins should carefully calibrate the levels of reporter overexpression. PCR tagging of endogenous proteins is, in principle, a preferable alternative.

Given the fast, cloning-free generation of donor DNA templates, the flexibility of design enabling protein or cellular expression analyses irrespective of gene size, relatively inexpensive reagent costs and high efficiency already in F0 animals, we propose that CRISPR-Cas9-assisted PCR tagging will become a standard method for generating precise zebrafish knock-in lines, expanding the genetic toolkit available in this model organism (see step-by-step protocol in Supplementary Materials and Methods).

## MATERIALS AND METHODS

### Zebrafish husbandry

Zebrafish were maintained under standard conditions (Westerfield, 2000) in the Bioresearch & Veterinary Services Aquatics facility at the Queen's Medical Research Institute, University of Edinburgh. Studies were carried out with approval from the UK Home Office under project license PP5258250. Adults were kept in a 14 h light/10 h dark cycle. Embryos were kept at 28.5°C in 10 mM HEPES-buffered E3 medium (5 mM NaCl, 0.17 mM KCl, 0.33 mM CaCl_2_, 0.33 mM MgSO_4_) or conditioned aquarium water with Methylene Blue. Embryos were staged according to dpf (Kimmel et al., 1995) and analyzed up to 5 dpf, before sexual differentiation. Wild-type zebrafish of the AB and WIK strains were used for the knock-in experiments described in this paper. The following established transgenic lines were used in this study: Tg(mnx1:GFP) (Flanagan-Steet et al., 2005) and Tg(mnx1:Gal4) (Wyart et al., 2009). The following knock-in reporter lines were generated in this study: *vamp1*^−mRuby3 KI^; *vamp2*^−mCherry KI^; *vamp3*^−mRuby3 KI^.

### CRISPR reagents

A step-by-step protocol is provided in the supplementary Materials and Methods. CRISPR crRNAs were designed with predicted cut sites as close as possible (≤12 bp) to the desired start or stop codons (see Table S3 for further details). CRISPR crRNAs were obtained from IDT DNA (Alt-R^®^ CRISPR-Cas9 crRNA, 2 nmol) and hybridized to universal Alt-R^®^ CRISPR-Cas9 tracrRNA to form 20 µM sgRNAs by heating to 95°C for 5 min and cooling gradually to room temperature. Engen Spy Cas9 enzyme (New England Biolabs) was used (see ‘PCR tagging injection mix preparation’ section). For optimization experiments in Fig. 2, to increase reproducibility between injection rounds, a large volume of 2× RNP stock was prepared with sgRNA and Cas9 each at 6 µM. RNP activity was tested by PCR amplification of the genomic region surrounding the target site using the primers indicated in Table S3, followed by restriction enzyme digest as indicated in Fig. S1.

### HDR template design

Oligonucleotide primer sequences used for HDR template synthesis are indicated and annotated in Table S3, with the following design considerations: for C-terminal knock-ins, a GGGGS linker sequence was used downstream of the endogenous protein and upstream of the fluorescent reporter, the reporter start codon was omitted, and the endogenous stop codon and untranslated region used in the right homology arm. For N-terminal knock-ins, the reporter sequence was used immediately downstream of the endogenous start codon (which could be partial and split over two exons); a GGGGS linker sequence was used downstream of the reporter and upstream of the remaining endogenous protein coding sequence. For cellular expression tagging, the sequence for membrane-tethered (N-terminal Fyn myristoylation motif, ‘mem’) mem-mEGFP or memmScarlet followed by the P2A peptide sequence were knocked-in at the N-terminal encoding end of the endogenous gene, without linker sequences. Where needed, primer sequences were designed to disrupt the PAM sequence in the HDR template to prevent repeated gRNA/Cas9 RNP cleavage. If this fell within the coding sequence, synonymous alterations were used. Modified primers consisted of 5′-end biotin and phosphorothioate bonds in the first 3-5 nucleotides in the 5′-end. All primers were obtained from IDT DNA (25-100 nmole scale, desalted). Further details can be found in the supplementary Materials and Methods.

### HDR template synthesis

HDR templates were produced by high-fidelity PCR using Phusion or Q5 polymerases (New England Biolabs) according to the supplier's instructions, using 35 amplification cycles in a final volume of 50-100 µl. PCR templates consisted of existing plasmids containing FP coding sequences: pCS2+mCherry; pCS2+mEGFP (containing the A206K monomerizing mutation); p3E-mRuby3; pME-memGFP-P2A (Addgene plasmid #80809); pTol2-mbp:memmScarlet-P2A-NKCC1a. PCR products were electrophoresed on 1% agarose gels and purified using the Monarch DNA Gel Extraction Kit (New England Biolabs); or alternatively treated with DpnI and ExoI and purified without prior electrophoresis using the Binding buffer with the Monarch DNA Gel Extraction Kit. In both cases products were eluted in 6 µl of water to maximize the concentration of the template. Further details can be found in the supplementary Materials and Methods.

### PCR tagging injection mix preparation

A step-by-step protocol is provided in the supplementary Materials and Methods. Injection solutions consisted of 3-4 µM sgRNA, 3-3.2 µM Engen Spy Cas9 enzyme (for optimization experiments, these were previously prepared as a large stock of 2× concentrated RNP), 10-125 ng/µl of HDR template and unless, otherwise indicated (Fig. 2; Fig. S4), small-molecule HDR modulators or 1% DMSO vehicle control were added at the following concentrations: 73 µM NU7441 (Stratech), 50 µM RS-1 (Merck Sigma-Aldrich), 50 µM L755507 (Stratech) or 7 µM HDR enhancer v2 (IDT DNA), in a final concentration of 1% DMSO. Injection solutions were prepared in a final volume of 3-10 µl and incubated at 37°C for 10 min. For optimization experiments, 2× RNP solution was diluted 1:2 with added HDR template for each injection condition, then incubated at 37°C for 10 min. The 2× stock and 1× prepared injection mixes were kept at 4°C and reused throughout the optimization experiments.

### Overexpression construct

To generate the Tol2 expression construct mEGFP-vamp1-10xUAS-vamp2-mCherry, we recombined 10 fmol each of a 5′-entry vector containing the mEGFP sequence at the N terminus of the zebrafish *vamp1* coding sequence with the entire cassette in a reverse orientation, a middle-entry vector containing 10 (palindromic) UAS repeats flanked by two minimal promoters, a 3′-entry vector containing the zebrafish *vamp2* coding sequence fused in frame to the mCherry coding sequence and 20 fmol of destination vector pDestTol2pA2 from the tol2kit (Kwan et al., 2007) in a LR reaction with LR Clonase II Plus (Thermo Fisher Scientific). Then, 3-4 clones were tested for correct recombination in a ‘Janus’ configuration (Distel et al., 2010) by digestion with restriction enzymes.

### Microinjection

Injection solutions were kept on ice following the 37°C incubation. One-cell stage WIK or AB eggs were injected with 1 nl of PCR tagging injection mix directly into the cell or within the yolk as close as possible to the cell. Eggs past the one-cell stage were not injected. Fertilized embryos were sorted at the end of the injection day, and then raised at 28.5°C until 3-5 dpf, when they were screened for normal development and fluorescence.

### Microscopy

Embryos that developed with a normal morphology to 3-5 dpf were first anesthetized with 600 µM tricaine (3-amino benzoic acid ethyl ester, Sigma-Aldrich) and mounted on their sides in 1.5% low-melting point agarose under a glass coverslip, and examined individually for fluorescence along their brain and spine, using a Zeiss AxioImager A1 equipped with a Plan Apochromat 20×/0.8NA objective and a HXP120 fluorescent light source. Embryos were scored for sparse, widespread or no fluorescence (see examples in Fig. 1), and investigators were unaware of experimental condition. At least three injection rounds were analyzed for optimization experiments in Fig. 2 (average 86 larvae per round). For light-sectioned high-resolution imaging of sparse or widespread F0 or F1-F2 zebrafish, larvae were mounted as above and imaged using a Zeiss LSM880 with Airyscan confocal and 488 nm (EGFP), 568 nm (mRuby3) and 594 nm (mCherry) laser lines, equipped with a Zeiss Plan-Apochromat 20×/0.8NA dry objective or Zeiss W Plan-Apochromat 20×/1.0NA water-dipping objective. In some cases, a Zeiss AxioImager Z1 equipped with an Apotome2 and 20×/0.8NA dry objective was used. Fiji/ImageJ was used for global adjustments of brightness and contrast and fluorescence intensity measurements. For NMJ analyses (Fig. 7) in PCR-tagged and Vamp1/2-FP overexpressing motor neurons, acquisition settings were identical. Using maximum intensity projections of axon terminals, a 5 pixel-thick segmented line was drawn along the centre of axon to capture 7-15 boutons per axon, and the GFP and mCherry fluorescence intensity (‘mean grey value’) along the line measured. For bouton heterogeneity analysis, intensities for each profile were normalized such that zero was set as the minimum grey value and 1 the maximum grey value in each channel (e.g. Fig. 7C). Maxima along the profiles (corresponding to synaptic terminals/boutons) were manually scored for expression of mCherry, EGFP or both (a total of 58 and 42 boutons were analyzed respectively in PCR tagged or overexpressing axons). For analyses of GFP/mCherry intensity outside of boutons (Fig. 7D), the bottom 10% of the total range of fluorescence intensity/grey value was determined for each axonal profile, corresponding to the minima along the profile. The average grey value of the bottom 10% for each channel was then determined (in arbitrary, non-normalized units). For figures, individual *z*-slices or maximum-intensity projections of *z*-stacks were made, and a representative *x*-*y* area was cropped. Figure panels were produced using Fiji and Adobe Illustrator. All zebrafish images and movies represent a lateral view of the spinal cord, anterior to the left and dorsal on top.

### Germline transmission

F0 larvae were raised to adulthood and at least 100 F1 offspring from outcrosses with wild-type strains were analyzed for a determination of whether the F0 adult was a founder. Number of adult F0 animals screened, number that were founders, and number of F1 offspring from each founder that carried a knock-in allele are described in Table S1.

### Knock-in junction analysis

A step-by-step protocol is provided in the supplementary Materials and Methods. F0 or F1 animals were genotyped by first extracting genomic DNA from whole embryos at 3-5 dpf using the HOTSHOT method (Meeker et al., 2007) or the Monarch Genomic DNA Purification Kit (New England Biolabs) to isolate high-molecular weight genomic DNA. PCR was then performed using Onetaq or Q5 (New England Biolabs) with the primer pairs indicated in Table S3 and according to the manufacturer's instructions, typically using 35-40 amplification cycles. For this analysis, locus-specific genotyping primers were located outside of the region used for the homology arms in the HDR template. PCR products were electrophoresed in 1-2% agarose gels. Products from F0 and F1 genotyping were purified with the Monarch Gel Extraction Kit (New England Biolabs) and verified by Sanger sequencing (Source Bioscience) with the PCR primers.

### Statistical analyses

All graphing and statistical tests were carried out using GraphPad Prism 9. Throughout the figures, error bars indicate standard deviation or 95% confidence intervals (assuming binomial distribution, calculated using the Wilson/Brown method) as indicated in the legend. Sample sizes (*n*) for each injection round and details of the statistical tests are indicated in the figure legends and provided in the tables in Table S2. Significant *P*-values are indicated as follows: *P<0.05, **P<0.01 or ***P<0.001.

## Supplementary Material

10.1242/develop.201323_sup1 Supplementary information

## References

[DEV201323C1] Aksoy, Y. A., Nguyen, D. T., Chow, S., Chung, R. S., Guillemin, G. J., Cole, N. J. and Hesselson, D. (2019). Chemical reprogramming enhances homology-directed genome editing in zebrafish embryos. *Commun. Biol.* 2, 198. 10.1038/s42003-019-0444-0 31149642 PMC6533270

[DEV201323C2] Archer, B. T., Ozçelik, T., Jahn, R., Francke, U. and Südhof, T. C. (1990). Structures and chromosomal localizations of two human genes encoding synaptobrevins 1 and 2. *J. Biol. Chem.* 265, 17267-17273. 10.1016/S0021-9258(17)44898-8 1976629

[DEV201323C3] Bhattacharya, S., Stewart, B. A., Niemeyer, B. A., Burgess, R. W., Mccabe, B. D., Lin, P., Boulianne, G., O'kane, C. J. and Schwarz, T. L. (2002). Members of the synaptobrevin/vesicle-associated membrane protein (VAMP) family in Drosophila are functionally interchangeable in vivo for neurotransmitter release and cell viability. *Proc. Natl. Acad. Sci. USA* 99, 13867-13872. 10.1073/pnas.202335999 12364587 PMC129789

[DEV201323C4] Boel, A., De Saffel, H., Steyaert, W., Callewaert, B., De Paepe, A., Coucke, P. J. and Willaert, A. (2018). CRISPR/Cas9-mediated homology-directed repair by ssODNs in zebrafish induces complex mutational patterns resulting from genomic integration of repair-template fragments. *Dis. Model. Mech.* 11, dmm035352. 10.1242/dmm.035352 30355591 PMC6215429

[DEV201323C5] Borisovska, M., Zhao, Y., Tsytsyura, Y., Glyvuk, N., Takamori, S., Matti, U., Rettig, J., Südhof, T. and Bruns, D. (2005). v-SNAREs control exocytosis of vesicles from priming to fusion. *EMBO J.* 24, 2114-2126. 10.1038/sj.emboj.7600696 15920476 PMC1150890

[DEV201323C6] Bourassa, C. V., Meijer, I. A., Merner, N. D., Grewal, K. K., Stefanelli, M. G., Hodgkinson, K., Ives, E. J., Pryse-Phillips, W., Jog, M., Boycott, K. et al. (2012). VAMP1 mutation causes dominant hereditary spastic ataxia in Newfoundland families. *Am. J. Hum. Genet.* 91, 548-552. 10.1016/j.ajhg.2012.07.018 22958904 PMC3511983

[DEV201323C7] Bussmann, J. and Schulte-Merker, S. (2011). Rapid BAC selection for tol2-mediated transgenesis in zebrafish. *Development* 138, 4327-4332. 10.1242/dev.068080 21865323

[DEV201323C8] Ceccaldi, R., Rondinelli, B. and D'andrea, A. D. (2016). Repair pathway choices and consequences at the double-strand break. *Trends Cell Biol.* 26, 52-64. 10.1016/j.tcb.2015.07.009 26437586 PMC4862604

[DEV201323C9] Chen, J., Poskanzer, K. E., Freeman, M. R. and Monk, K. R. (2020). Live-imaging of astrocyte morphogenesis and function in zebrafish neural circuits. *Nat. Neurosci.* 23, 1297-1306. 10.1038/s41593-020-0703-x 32895565 PMC7530038

[DEV201323C10] Cong, L., Ran, F. A., Cox, D., Lin, S., Barretto, R., Habib, N., Hsu, P. D., Wu, X., Jiang, W., Marraffini, L. A. et al. (2013). Multiplex genome engineering using CRISPR/Cas systems. *Science* 339, 819-823. 10.1126/science.1231143 23287718 PMC3795411

[DEV201323C11] Culp, P., Nüsslein-Volhard, C. and Hopkins, N. (1991). High-frequency germ-line transmission of plasmid DNA sequences injected into fertilized zebrafish eggs. *Proc. Natl. Acad. Sci. USA* 88, 7953-7957. 10.1073/pnas.88.18.7953 1910170 PMC52423

[DEV201323C12] Danner, E., Bashir, S., Yumlu, S., Wurst, W., Wefers, B. and Kühn, R. (2017). Control of gene editing by manipulation of DNA repair mechanisms. *Mamm. Genome* 28, 262-274. 10.1007/s00335-017-9688-5 28374058

[DEV201323C13] Distel, M., Hocking, J. C., Volkmann, K. and Köster, R. W. (2010). The centrosome neither persistently leads migration nor determines the site of axonogenesis in migrating neurons in vivo. *J. Cell Biol.* 191, 875-890. 10.1083/jcb.201004154 21059852 PMC2983064

[DEV201323C14] Driever, W., Solnica-Krezel, L., Schier, A. F., Neuhauss, S. C., Malicki, J., Stemple, D. L., Stainier, D. Y., Zwartkruis, F., Abdelilah, S., Rangini, Z. et al. (1996). A genetic screen for mutations affecting embryogenesis in zebrafish. *Development* 123, 37-46. 10.1242/dev.123.1.37 9007227

[DEV201323C15] Early, J. J., Cole, K. L., Williamson, J. M., Swire, M., Kamadurai, H., Muskavitch, M. and Lyons, D. A. (2018). An automated high-resolution in vivo screen in zebrafish to identify chemical regulators of myelination. *eLife* 7, e35136. 10.7554/eLife.35136 29979149 PMC6056238

[DEV201323C16] Feldmann, A., Winterstein, C., White, R., Trotter, J. and Krämer-Albers, E.-M. (2009). Comprehensive analysis of expression, subcellular localization, and cognate pairing of SNARE proteins in oligodendrocytes. *J. Neurosci. Res.* 87, 1760-1772. 10.1002/jnr.22020 19185015

[DEV201323C17] Feldmann, A., Amphornrat, J., Schönherr, M., Winterstein, C., Möbius, W., Ruhwedel, T., Danglot, L., Nave, K.-A., Galli, T., Bruns, D. et al. (2011). Transport of the major myelin proteolipid protein is directed by VAMP3 and VAMP7. *J. Neurosci.* 31, 5659-5672. 10.1523/JNEUROSCI.6638-10.2011 21490207 PMC6622839

[DEV201323C18] Feng, S., Wang, Z., Li, A., Xie, X., Liu, J., Li, S., Li, Y., Wang, B., Hu, L., Yang, L. et al. (2021). Strategies for high-efficiency mutation using the CRISPR/Cas system. *Front. Cell Dev. Biol.* 9, 803252. 10.3389/fcell.2021.803252 35198566 PMC8860194

[DEV201323C19] Flanagan-Steet, H., Fox, M. A., Meyer, D. and Sanes, J. R. (2005). Neuromuscular synapses can form in vivo by incorporation of initially aneural postsynaptic specializations. *Development* 132, 4471-4481. 10.1242/dev.02044 16162647

[DEV201323C20] Fueller, J., Herbst, K., Meurer, M., Gubicza, K., Kurtulmus, B., Knopf, J. D., Kirrmaier, D., Buchmuller, B. C., Pereira, G., Lemberg, M. K. et al. (2020). CRISPR-Cas12a-assisted PCR tagging of mammalian genes. *J. Cell Biol.* 219, e201910210. 10.1083/jcb.201910210 32406907 PMC7265327

[DEV201323C21] Gordon, S. L., Harper, C. B., Smillie, K. J. and Cousin, M. A. (2016). A fine balance of synaptophysin levels underlies efficient retrieval of synaptobrevin II to synaptic vesicles. *PLoS One* 11, e0149457. 10.1371/journal.pone.0149457 26871701 PMC4752265

[DEV201323C22] Grote, E., Hao, J. C., Bennett, M. K. and Kelly, R. B. (1995). A targeting signal in VAMP regulating transport to synaptic vesicles. *Cell* 81, 581-589. 10.1016/0092-8674(95)90079-9 7758112

[DEV201323C23] Gutierrez-Triana, J. A., Tavhelidse, T., Thumberger, T., Thomas, I., Wittbrodt, B., Kellner, T., Anlas, K., Tsingos, E. and Wittbrodt, J. (2018). Efficient single-copy HDR by 5’ modified long dsDNA donors. *eLife* 7, e39468. 10.7554/eLife.39468 30156184 PMC6125127

[DEV201323C24] Haffter, P., Granato, M., Brand, M., Mullins, M. C., Hammerschmidt, M., Kane, D. A., Odenthal, J., Van Eeden, F. J., Jiang, Y. J., Heisenberg, C. P. et al. (1996). The identification of genes with unique and essential functions in the development of the zebrafish, Danio rerio. *Development* 123, 1-36. 10.1242/dev.123.1.1 9007226

[DEV201323C25] Halpern, M. E., Rhee, J., Goll, M. G., Akitake, C. M., Parsons, M. and Leach, S. D. (2008). Gal4/UAS transgenic tools and their application to zebrafish. *Zebrafish* 5, 97-110. 10.1089/zeb.2008.0530 18554173 PMC6469517

[DEV201323C26] Hisano, Y., Sakuma, T., Nakade, S., Ohga, R., Ota, S., Okamoto, H., Yamamoto, T. and Kawahara, A. (2015). Precise in-frame integration of exogenous DNA mediated by CRISPR/Cas9 system in zebrafish. *Sci. Rep.* 5, 8841. 10.1038/srep08841 25740433 PMC4350073

[DEV201323C27] Hoshijima, K., Jurynec, M. J. and Grunwald, D. J. (2016). Precise editing of the zebrafish genome made simple and efficient. *Dev. Cell* 36, 654-667. 10.1016/j.devcel.2016.02.015 27003937 PMC4806538

[DEV201323C28] Hoshijima, K., Jurynec, M. J., Klatt Shaw, D., Jacobi, A. M., Behlke, M. A. and Grunwald, D. J. (2019). Highly efficient CRISPR-Cas9-based methods for generating deletion mutations and F0 embryos that lack gene function in Zebrafish. *Dev. Cell* 51, 645-657.e4. 10.1016/j.devcel.2019.10.004 31708433 PMC6891219

[DEV201323C29] Howe, K., Clark, M. D., Torroja, C. F., Torrance, J., Berthelot, C., Muffato, M., Collins, J. E., Humphray, S., Mclaren, K., Matthews, L. et al. (2013). The zebrafish reference genome sequence and its relationship to the human genome. *Nature* 496, 498-503. 10.1038/nature12111 23594743 PMC3703927

[DEV201323C30] Hwang, W. Y., Fu, Y., Reyon, D., Maeder, M. L., Tsai, S. Q., Sander, J. D., Peterson, R. T., Yeh, J.-R. J. and Joung, J. K. (2013). Efficient genome editing in zebrafish using a CRISPR-Cas system. *Nat. Biotechnol.* 31, 227-229. 10.1038/nbt.2501 23360964 PMC3686313

[DEV201323C31] Jao, L.-E., Wente, S. R. and Chen, W. (2013). Efficient multiplex biallelic zebrafish genome editing using a CRISPR nuclease system. *Proc. Natl. Acad. Sci. USA* 110, 13904-13909. 10.1073/pnas.1308335110 23918387 PMC3752207

[DEV201323C32] Jinek, M., Chylinski, K., Fonfara, I., Hauer, M., Doudna, J. A. and Charpentier, E. (2012). A programmable dual-RNA-guided DNA endonuclease in adaptive bacterial immunity. *Science* 337, 816-821. 10.1126/science.1225829 22745249 PMC6286148

[DEV201323C33] Kanca, O., Zirin, J., Garcia-Marques, J., Knight, S. M., Yang-Zhou, D., Amador, G., Chung, H., Zuo, Z., Ma, L., He, Y. et al. (2019). An efficient CRISPR-based strategy to insert small and large fragments of DNA using short homology arms. *eLife* 8, e51539. 10.7554/eLife.51539 31674908 PMC6855806

[DEV201323C34] Kawakami, K. (2007). Tol2: a versatile gene transfer vector in vertebrates. *Genome Biol.* 8 Suppl 1, S7. 10.1186/gb-2007-8-s1-s7 18047699 PMC2106836

[DEV201323C35] Keatinge, M., Tsarouchas, T. M., Munir, T., Porter, N. J., Larraz, J., Gianni, D., Tsai, H.-H., Becker, C. G., Lyons, D. A. and Becker, T. (2021). CRISPR gRNA phenotypic screening in zebrafish reveals pro-regenerative genes in spinal cord injury. *PLoS Genet.* 17, e1009515. 10.1371/journal.pgen.1009515 33914736 PMC8084196

[DEV201323C36] Kim, J. H., Lee, S.-R., Li, L.-H., Park, H.-J., Park, J.-H., Lee, K. Y., Kim, M.-K., Shin, B. A. and Choi, S.-Y. (2011). High cleavage efficiency of a 2A peptide derived from porcine teschovirus-1 in human cell lines, zebrafish and mice. *PLoS One* 6, e18556. 10.1371/journal.pone.0018556 21602908 PMC3084703

[DEV201323C37] Kimmel, C. B., Ballard, W. W., Kimmel, S. R., Ullmann, B. and Schilling, T. F. (1995). Stages of embryonic development of the zebrafish. *Dev. Dyn.* 203, 253-310. 10.1002/aja.1002030302 8589427

[DEV201323C38] Kosuta, C., Daniel, K., Johnstone, D. L., Mongeon, K., Ban, K., Leblanc, S., Macleod, S., Et-Tahiry, K., Ekker, M., Mackenzie, A. et al. (2018). High-throughput DNA extraction and genotyping of 3dpf zebrafish larvae by fin clipping. *J. Vis. Exp.* 136, 58024. 10.3791/58024 PMC6102016 30010654

[DEV201323C39] Kroll, F., Powell, G. T., Ghosh, M., Gestri, G., Antinucci, P., Hearn, T. J., Tunbak, H., Lim, S., Dennis, H. W., Fernandez, J. M. et al. (2021). A simple and effective F0 knockout method for rapid screening of behaviour and other complex phenotypes. *eLife* 10, e59683. 10.7554/eLife.59683 33416493 PMC7793621

[DEV201323C40] Kwan, K. M., Fujimoto, E., Grabher, C., Mangum, B. D., Hardy, M. E., Campbell, D. S., Parant, J. M., Yost, H. J., Kanki, J. P. and Chien, C.-B. (2007). The Tol2kit: a multisite gateway-based construction kit for Tol2 transposon transgenesis constructs. *Dev. Dyn.* 236, 3088-3099. 10.1002/dvdy.21343 17937395

[DEV201323C41] Lam, M., Takeo, K., Almeida, R. G., Cooper, M. H., Wu, K., Iyer, M., Kantarci, H. and Zuchero, J. B. (2022). CNS myelination requires VAMP2/3-mediated membrane expansion in oligodendrocytes. *Nat. Commun.* 13, 5583. 10.1038/s41467-022-33200-4 36151203 PMC9508103

[DEV201323C42] Liu, Y., Sugiura, Y. and Lin, W. (2011). The role of synaptobrevin1/VAMP1 in Ca2+-triggered neurotransmitter release at the mouse neuromuscular junction. *J. Physiol. (Lond.)* 589, 1603-1618. 10.1113/jphysiol.2010.201939 21282288 PMC3099018

[DEV201323C43] Liu, Z., Chen, O., Wall, J. B. J., Zheng, M., Zhou, Y., Wang, L., Vaseghi, H. R., Qian, L. and Liu, J. (2017). Systematic comparison of 2A peptides for cloning multi-genes in a polycistronic vector. *Sci. Rep.* 7, 2193. 10.1038/s41598-017-02460-2 28526819 PMC5438344

[DEV201323C44] Liu, Y., Sugiura, Y., Südhof, T. C. and Lin, W. (2019). Ablation of All Synaptobrevin vSNAREs Blocks Evoked But Not Spontaneous Neurotransmitter Release at Neuromuscular Synapses. *J. Neurosci.* 39, 6049-6066. 10.1523/JNEUROSCI.0403-19.2019 31160536 PMC6668203

[DEV201323C45] Liu, F., Kambakam, S., Almeida, M. P., Ming, Z., Welker, J. M., Wierson, W. A., Schultz-Rogers, L. E., Ekker, S. C., Clark, K. J., Essner, J. J. et al. (2022). Cre/lox regulated conditional rescue and inactivation with zebrafish UFlip alleles generated by CRISPR-Cas9 targeted integration. *eLife* 11, e71478. 10.7554/eLife.71478 35713402 PMC9270027

[DEV201323C46] Marques, S., Zeisel, A., Codeluppi, S., Van Bruggen, D., Mendanha Falcão, A., Xiao, L., Li, H., Häring, M., Hochgerner, H., Romanov, R. A. et al. (2016). Oligodendrocyte heterogeneity in the mouse juvenile and adult central nervous system. *Science* 352, 1326-1329. 10.1126/science.aaf6463 27284195 PMC5221728

[DEV201323C47] Mcmahon, H. T., Ushkaryov, Y. A., Edelmann, L., Link, E., Binz, T., Niemann, H., Jahn, R. and Südhof, T. C. (1993). Cellubrevin is a ubiquitous tetanus-toxin substrate homologous to a putative synaptic vesicle fusion protein. *Nature* 364, 346-349. 10.1038/364346a0 8332193

[DEV201323C48] Meeker, N. D., Hutchinson, S. A., Ho, L. and Trede, N. S. (2007). Method for isolation of PCR-ready genomic DNA from zebrafish tissues. *BioTechniques* 43, 610, 612, 614. 10.2144/000112619 18072590

[DEV201323C49] Mi, J. and Andersson, O. (2023). Efficient knock-in method enabling lineage tracing in zebrafish. *Life Sci. Alliance* 6, e202301944. 10.26508/lsa.202301944 36878640 PMC9990459

[DEV201323C50] Mosimann, C., Puller, A.-C., Lawson, K. L., Tschopp, P., Amsterdam, A. and Zon, L. I. (2013). Site-directed zebrafish transgenesis into single landing sites with the phiC31 integrase system. *Dev. Dyn.* 242, 949-963. 10.1002/dvdy.23989 23723152 PMC3775328

[DEV201323C51] Murakami, Y. and Kobayashi, T. (2022). An effective double gene knock-in strategy using small-molecule L755507 in the medaka fish (Oryzias latipes). *Genesis* 60, e23465. 10.1002/dvg.23465 35072325

[DEV201323C52] Nystuen, A. M., Schwendinger, J. K., Sachs, A. J., Yang, A. W. and Haider, N. B. (2007). A null mutation in VAMP1/synaptobrevin is associated with neurological defects and prewean mortality in the lethal-wasting mouse mutant. *Neurogenetics* 8, 1-10. 10.1007/s10048-006-0068-7 17102983

[DEV201323C53] Parlati, F., Mcnew, J. A., Fukuda, R., Miller, R., Söllner, T. H. and Rothman, J. E. (2000). Topological restriction of SNARE-dependent membrane fusion. *Nature* 407, 194-198. 10.1038/35025076 11001058

[DEV201323C54] Peng, L., Adler, M., Demogines, A., Borrell, A., Liu, H., Tao, L., Tepp, W. H., Zhang, S.-C., Johnson, E. A., Sawyer, S. L. et al. (2014). Widespread sequence variations in VAMP1 across vertebrates suggest a potential selective pressure from botulinum neurotoxins. *PLoS Pathog.* 10, e1004177. 10.1371/journal.ppat.1004177 25010769 PMC4092145

[DEV201323C55] Perucho, M., Hanahan, D. and Wigler, M. (1980). Genetic and physical linkage of exogenous sequences in transformed cells. *Cell* 22, 309-317. 10.1016/0092-8674(80)90178-6 6253083

[DEV201323C56] Ranawakage, D. C., Okada, K., Sugio, K., Kawaguchi, Y., Kuninobu-Bonkohara, Y., Takada, T. and Kamachi, Y. (2020). Efficient CRISPR-Cas9-mediated knock-in of composite tags in zebrafish using long ssDNA as a donor. *Front. Cell Dev. Biol.* 8, 598634. 10.3389/fcell.2020.598634 33681181 PMC7928300

[DEV201323C57] Roberts, J. A., Miguel-Escalada, I., Slovik, K. J., Walsh, K. T., Hadzhiev, Y., Sanges, R., Stupka, E., Marsh, E. K., Balciuniene, J., Balciunas, D. et al. (2014). Targeted transgene integration overcomes variability of position effects in zebrafish. *Development* 141, 715-724. 10.1242/dev.100347 24449846 PMC3899822

[DEV201323C58] Salpietro, V., Lin, W., Delle Vedove, A., Storbeck, M., Liu, Y., Efthymiou, S., Manole, A., Wiethoff, S., Ye, Q., Saggar, A. et al. (2017). Homozygous mutations in VAMP1 cause a presynaptic congenital myasthenic syndrome. *Ann. Neurol.* 81, 597-603. 10.1002/ana.24905 28253535 PMC5413866

[DEV201323C59] Salpietro, V., Malintan, N. T., Llano-Rivas, I., Spaeth, C. G., Efthymiou, S., Striano, P., Vandrovcova, J., Cutrupi, M. C., Chimenz, R., David, E. et al. (2019). Mutations in the neuronal vesicular SNARE VAMP2 affect synaptic membrane fusion and impair human neurodevelopment. *Am. J. Hum. Genet.* 104, 721-730. 10.1016/j.ajhg.2019.02.016 30929742 PMC6451933

[DEV201323C60] Schoch, S., Deák, F., Königstorfer, A., Mozhayeva, M., Sara, Y., Südhof, T. C. and Kavalali, E. T. (2001). SNARE function analyzed in synaptobrevin/VAMP knockout mice. *Science* 294, 1117-1122. 10.1126/science.1064335 11691998

[DEV201323C61] Schwarz, Y., Zhao, N., Kirchhoff, F. and Bruns, D. (2017). Astrocytes control synaptic strength by two distinct v-SNARE-dependent release pathways. *Nat. Neurosci.* 20, 1529-1539. 10.1038/nn.4647 28945220

[DEV201323C62] Schwinn, M. K., Steffen, L. S., Zimmerman, K., Wood, K. V. and Machleidt, T. (2020). A simple and scalable strategy for analysis of endogenous protein dynamics. *Sci. Rep.* 10, 8953. 10.1038/s41598-020-65832-1 32488146 PMC7265437

[DEV201323C63] Seleit, A., Aulehla, A. and Paix, A. (2021). Endogenous protein tagging in medaka using a simplified CRISPR/Cas9 knock-in approach. *eLife* 10, e75050. 10.7554/eLife.75050 34870593 PMC8691840

[DEV201323C64] Shin, J., Chen, J. and Solnica-Krezel, L. (2014). Efficient homologous recombination-mediated genome engineering in zebrafish using TALE nucleases. *Development* 141, 3807-3818. 10.1242/dev.108019 25249466 PMC4197590

[DEV201323C65] Sonoda, E., Hochegger, H., Saberi, A., Taniguchi, Y. and Takeda, S. (2006). Differential usage of non-homologous end-joining and homologous recombination in double strand break repair. *DNA Repair (Amst.)* 5, 1021-1029. 10.1016/j.dnarep.2006.05.022 16807135

[DEV201323C66] Stadler, C., Rexhepaj, E., Singan, V. R., Murphy, R. F., Pepperkok, R., Uhlén, M., Simpson, J. C. and Lundberg, E. (2013). Immunofluorescence and fluorescent-protein tagging show high correlation for protein localization in mammalian cells. *Nat. Methods* 10, 315-323. 10.1038/nmeth.2377 23435261

[DEV201323C67] Stratigopoulos, G., De Rosa, M. C., Leduc, C. A., Leibel, R. L. and Doege, C. A. (2018). DMSO increases efficiency of genome editing at two non-coding loci. *PLoS One* 13, e0198637. 10.1371/journal.pone.0198637 29864154 PMC5986138

[DEV201323C68] Taylor, J. S., Van de Peer, Y., Braasch, I. and Meyer, A. (2001). Comparative genomics provides evidence for an ancient genome duplication event in fish. *Philos. Trans. R. Soc. Lond. B, Biol. Sci.* 356, 1661-1679. 10.1098/rstb.2001.0975 11604130 PMC1088543

[DEV201323C69] Urbina, F. L. and Gupton, S. L. (2020). SNARE-mediated exocytosis in neuronal development. *Front. Mol. Neurosci.* 13, 133. 10.3389/fnmol.2020.00133 32848598 PMC7427632

[DEV201323C70] Usher, I., Ligammari, L., Ahrabi, S., Hepburn, E., Connolly, C., Bond, G. L., Flanagan, A. M. and Cottone, L. (2022). Optimizing crispr/cas9 editing of repetitive single nucleotide variants. *Front. Genome Ed.* 4, 932434. 10.3389/fgeed.2022.932434 35865001 PMC9294353

[DEV201323C71] Verhage, M. and Sørensen, J. B. (2020). Snareopathies: diversity in mechanisms and symptoms. *Neuron* 107, 22-37. 10.1016/j.neuron.2020.05.036 32559416

[DEV201323C72] Vuong, C. K., Wei, W., Lee, J.-A., Lin, C.-H., Damianov, A., De La Torre-Ubieta, L., Halabi, R., Otis, K. O., Martin, K. C., O'dell, T. J. et al. (2018). Rbfox1 regulates synaptic transmission through the inhibitory neuron-specific vSNARE Vamp1. *Neuron* 98, 127-141.e7. 10.1016/j.neuron.2018.03.008 29621484 PMC5890944

[DEV201323C73] Westerfield, M. (2000). The zebrafish book: a guide for the laboratory use of zebrafish (*Danio rerio*), 4th ed. University of Oregon Press.

[DEV201323C74] Wierson, W. A., Welker, J. M., Almeida, M. P., Mann, C. M., Webster, D. A., Torrie, M. E., Weiss, T. J., Kambakam, S., Vollbrecht, M. K., Lan, M. et al. (2020). Efficient targeted integration directed by short homology in zebrafish and mammalian cells. *eLife* 9, e53968. 10.7554/eLife.53968 32412410 PMC7228771

[DEV201323C75] Wyart, C., Del Bene, F., Warp, E., Scott, E. K., Trauner, D., Baier, H. and Isacoff, E. Y. (2009). Optogenetic dissection of a behavioural module in the vertebrate spinal cord. *Nature* 461, 407-410. 10.1038/nature08323 19759620 PMC2770190

[DEV201323C76] Yang, H., Ren, S., Yu, S., Pan, H., Li, T., Ge, S., Zhang, J. and Xia, N. (2020). Methods favoring homology-directed repair choice in response to CRISPR/Cas9 induced-double strand breaks. *Int. J. Mol. Sci.* 21, 6461. 10.3390/ijms21186461 32899704 PMC7555059

[DEV201323C77] Yu, Y., Guo, Y., Tian, Q., Lan, Y., Yeh, H., Zhang, M., Tasan, I., Jain, S. and Zhao, H. (2020). An efficient gene knock-in strategy using 5’-modified double-stranded DNA donors with short homology arms. *Nat. Chem. Biol.* 16, 387-390. 10.1038/s41589-019-0432-1 31873222 PMC7085973

[DEV201323C78] Zhang, Y., Chen, K., Sloan, S. A., Bennett, M. L., Scholze, A. R., O'keeffe, S., Phatnani, H. P., Guarnieri, P., Caneda, C., Ruderisch, N. et al. (2014). An RNA-sequencing transcriptome and splicing database of glia, neurons, and vascular cells of the cerebral cortex. *J. Neurosci.* 34, 11929-11947. 10.1523/JNEUROSCI.1860-14.2014 25186741 PMC4152602

[DEV201323C79] Zhang, X., Zhang, Z., Zhao, Q. and Lou, X. (2020). Rapid and efficient live zebrafish embryo genotyping. *Zebrafish* 17, 56-58. 10.1089/zeb.2019.1796 31851585

[DEV201323C80] Zimmermann, J., Trimbuch, T. and Rosenmund, C. (2014). Synaptobrevin 1 mediates vesicle priming and evoked release in a subpopulation of hippocampal neurons. *J. Neurophysiol.* 112, 1559-1565. 10.1152/jn.00340.2014 24944211

