# Supplementary material for: Fast, precise and cloning-free knock-in of reporter sequences in vivo with high efficiency
Source: Development. 2023 Jun 29;150(12):dev201323. doi: 10.1242/dev.201323 (PMC10323249; doi:10.1242/dev.201323)
Supplement: Supplementary information [file develop-150-201323-s1.pdf]

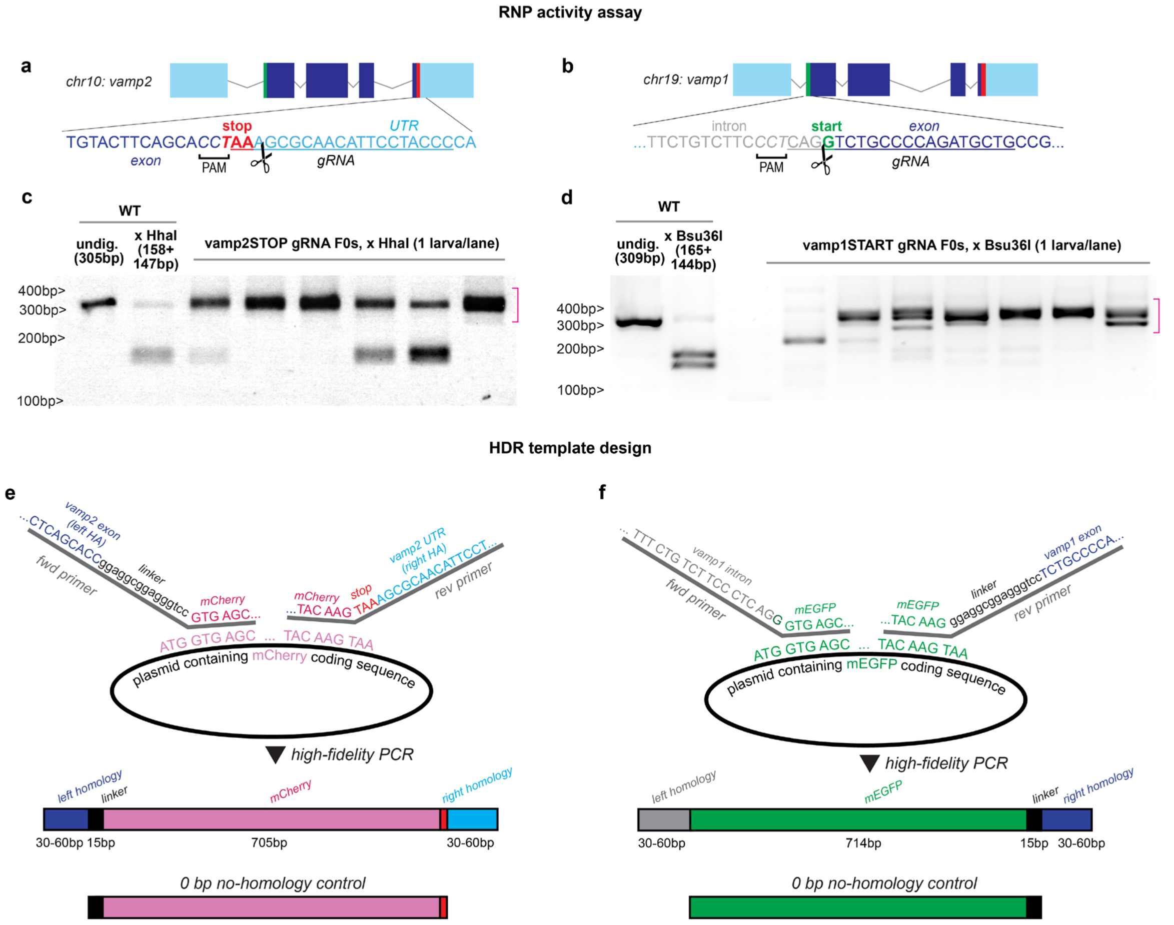

Fig. S1. Design of *vamp2* C-terminal and *vamp1* N-terminal tagging.

a-b. gRNA spacer sequence (underlined) targeting the C-terminus of *vamp2* (a) and N-terminus of *vamp1* (b). Start codon in green, stop codon in red, coding sequence in dark blue, untranslated sequence (UTR) in light blue, intronic sequence in grey. PAM in italics, scissors indicate cleavage site. **c-d.** High gRNA activity demonstrated by PCR from genomic DNA from F0 larvae injected with RNP around the target sites, followed by restriction digest. Wildtype (WT) genomic DNA produces a 305bp/309bp product cleaved by HhaI/Bsu36I, but in most F0 larvae indels resulting from high RNP activity disrupt cleavage (indicated by brackets). **e-f.** Diagram of cloning-free HDR template synthesis for *vamp2-mCherry* (e) and *mEGFP-vamp1* (f) tagging using high-fidelity PCR with primers that incorporate short homology arms, highlighting key sequences and their lengths.

Development • Supplementary information

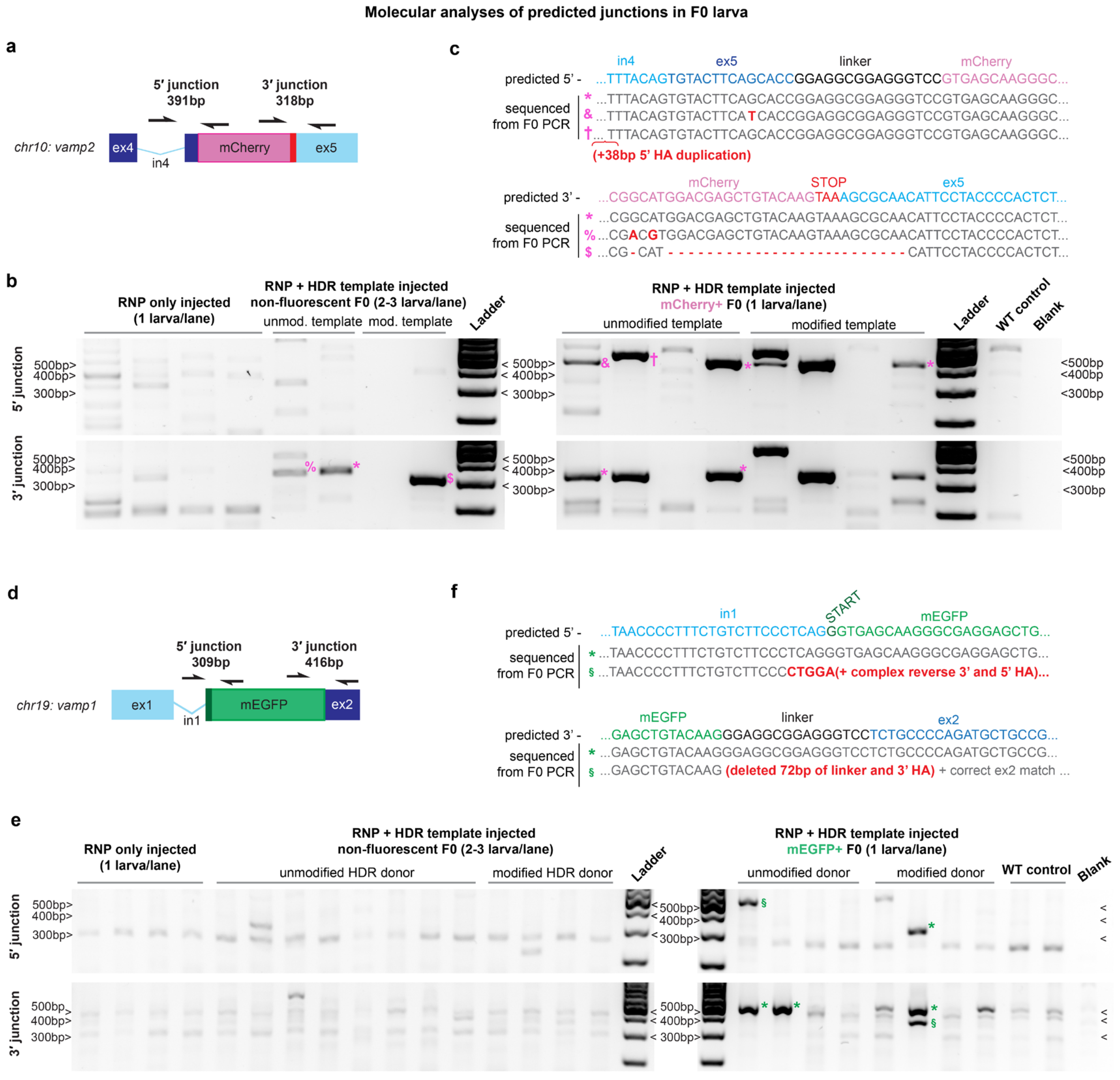

Fig. S2. Molecular analysis of predicted on-target integration in F0 injected larvae.

a. Primer design for analyses of 5′and 3′junctions of vamp2-mCherry tagging. b-c. PCR products of approximately the expected size were only amplified from F0 larvae injected with RNP and HDR template, but not from RNP-only injected larvae (b). Note non-fluorescent larvae injected with RNP and template could still exhibit on-target knock-in (indicated by % * and $ in 3′ junction). Some of these PCR products were sequenced (c) showing correct, precise integration (indicated by *) or imprecise integration with varying degrees of alterations (point mutations and indels in red, indicated by &, †, %, $). **d.** Primer design for analyses of 5′and 3′ junctions of *mEGFP-vamp1* tagging. **e-f.** PCR products of approximately the expected size were only amplified from F0 larvae injected with RNP and HDR template, but not from RNP-only injected larvae (e). Some of these PCR products were sequenced (f) showing correct, precise integration (indicated by *) or imprecise integration with varying degrees of alterations (e.g. indels indicated in red, bands indicated by §).

Development • Supplementary information

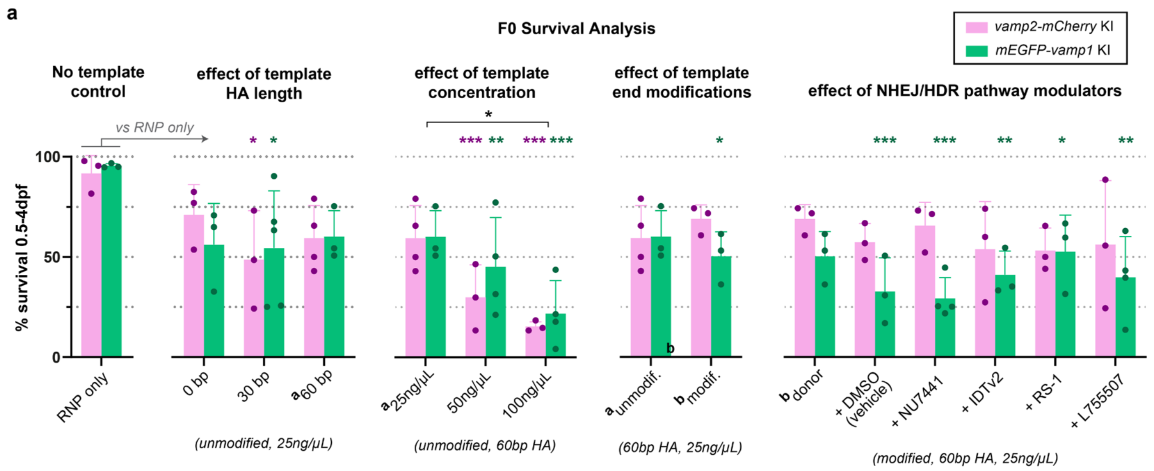

Fig. S3. PCR tagging F0 survival analysis, related to Fig 2.

a. Survival of fertilized injected embryos between 0 and 3-4dpf (only larvae without gross morphological abnormalities were analyzed). Note high survival of RNP only injected larvae but decreased survival with addition of HDR template to the injection mix. Note dose-dependent survival decrease with increasing template concentration. Circles indicate individual injection rounds and bars indicate the mean of 3-5 injection rounds per condition. Error bars indicate standard deviation. Asterisks indicate $p<0.05$ (*), <0.01(**) or <0.001(***) in two-way ANOVA corrected for multiple comparisons (for details see Supplementary File 2). For ease of comparison, data indicated by **a** and **b** are shown in more than one graph.

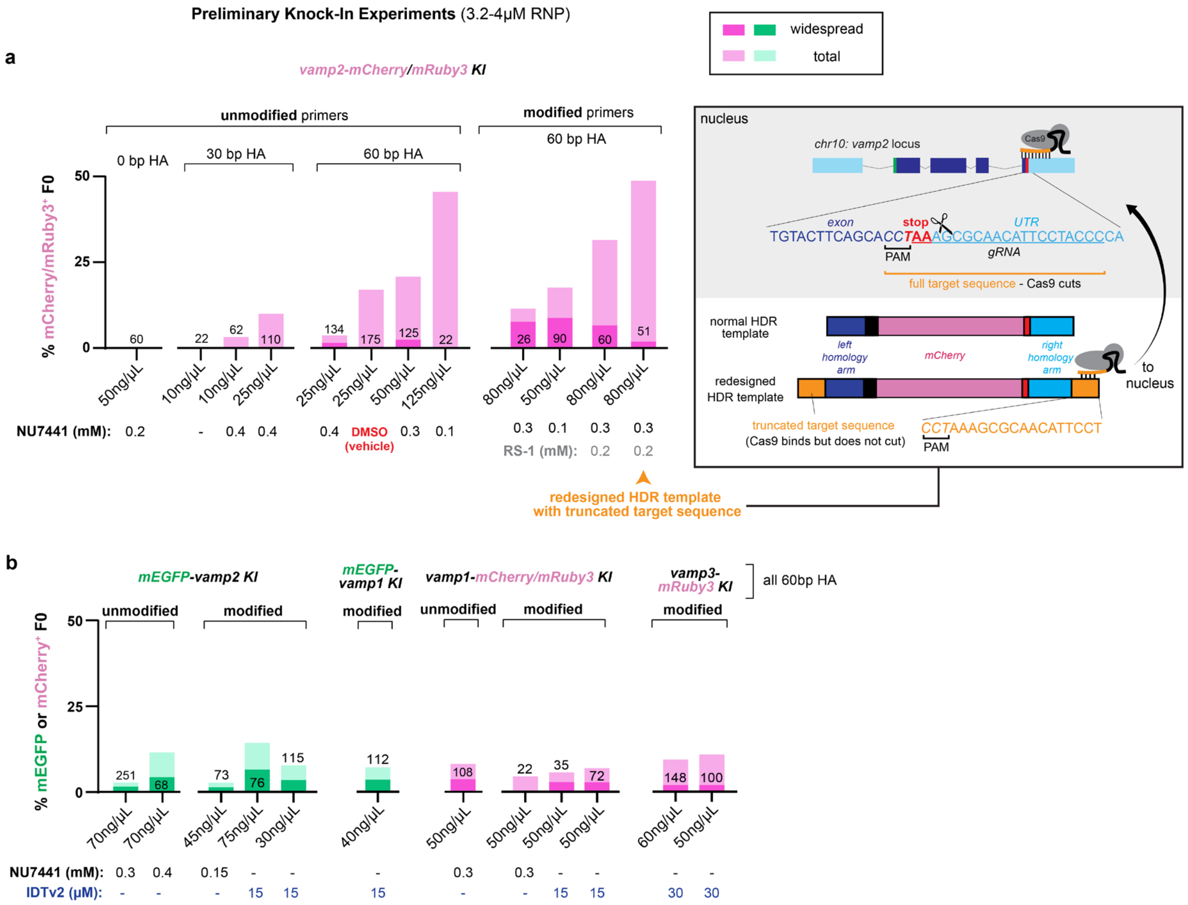

Fig. S4. Preliminary PCR tagging experiments exploring further parameter space and template design.

a. *vamp2-mCherry* or -*mRuby3* PCR tagging using slightly higher RNP concentrations, different HDR/NHEJ modulator concentrations (including in combination), and additional features in the HDR template designed to enrich its nuclear localization (inclusion of a truncated gRNA target sequence that allows binding but not cleavage by RNP). Note efficiency of *vamp2-mCherry* tagging approaches 50% in some conditions. **b.** As in a, relating to N-terminal *vamp2* tagging, and *vamp1* and *vamp3* tagging. In a and b, bars represent one injection round; template concentration indicated in x-axis ticks with HDR modulator concentrations below, the number of 3-5dpf F0 larvae analyzed is indicated in each bar.

Development • Supplementary information

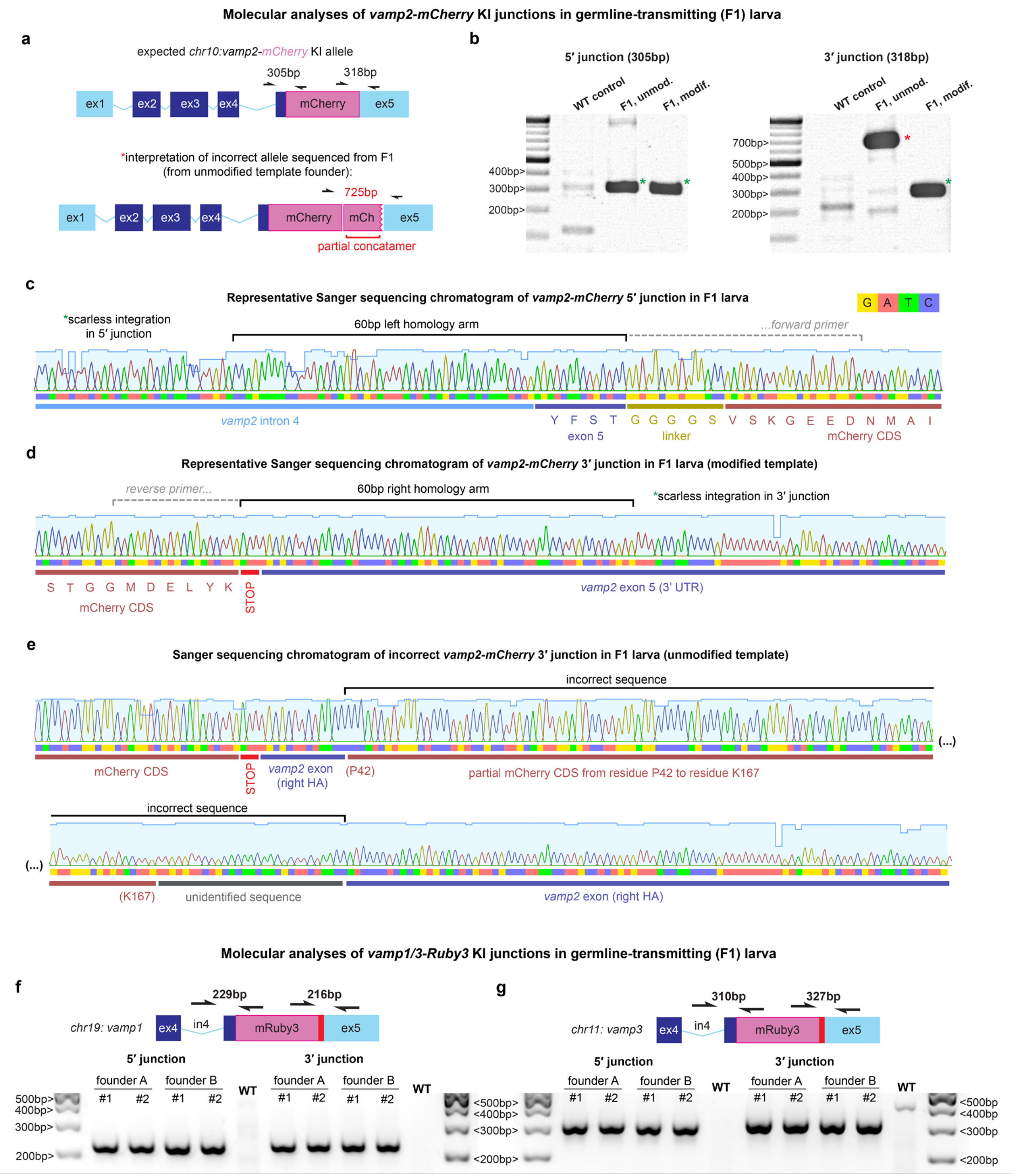

Fig. S5. Molecular analysis of knock-in alleles in F1 larvae.

a. Primer design for 5′ and 3′ *vamp2-mCherry* junction analysis. **b.** PCR products indicate junctions of predicted size in lines made with modified ("mod.") HDR templates, while line made with unmodified ("unmod.") template show incorrect longer 3′ junction (*). **c-d.** Example of Sanger sequencing of PCR products of correctly-sized 5′ (c) and 3′ junctions (d) showing scarless knock-in. Blue bars show base call quality score. CDS: coding sequence. **e.** Sequencing result of (incorrectly sized) PCR products of 3′ junction in F1 larva in line made with unmodified template, showing second partial mCherry CDS copy (compare with d). Suggests the incorrect allele shown in a. **f-g.** 5′ and 3′ junction analyses of *vamp1-mRuby3* (f) and *vamp3-mRuby3* (g) alleles in F1 larvae. Genomic DNA from two F1 animals (#1, #2) from each of two founders ('A' and 'B') were tested. WT= wildtype DNA control.

Development • Supplementary information

Table S1. Germline transmission of PCR tagged zebrafish.

Knock-in line	% F0 adult that are founders (with FP$^+$ F1)	% F1 offspring that is FP$^+$ from each F0 founder	Is F1 integration precise?
vamp2-mCherry (unmodified temp.)	1/17 of mCherry$^+$<br>0/12 of mCherry$^-$	• 11/252 (4%)	No (see Fig. S5)
(modified temp.)	1/4 of sparse mCherry$^+$<br>2/2 of widespread mCherry$^+$	• 7/485 (1%, sparse)<br>• 7/499 (1%, widespread)<br>• 129/362 (36%, widespread)	Yes, in all 3 (Fig. S5 & Sanger sequencing)
mEGFP-vamp2 (unmodified)	0/3 of widespread mEGFP$^+$	-	-
(modified)	0/2 of sparse mEGFP$^+$<br>0/1 of widespread mEGFP$^+$		
vamp1-mRuby3 (modified)	0/3 of sparse mRuby3$^+$<br>2/4 of widespread mRuby3$^+$	• 1/23 (4%, widespread)<br>• 37/186 (20%, widespread)	Yes, in both (Fig. S5 & Sanger sequencing)
vamp3-mRuby3 (modified)	0/7 of sparse mRuby3$^+$<br>2/3 of widespread mRuby3$^+$	• 14/28 (50%, widespread)<br>• 6/30 (20%, widespread)	Yes (Fig. S5 & Sanger sequencing)

Table S2. Statistical details

This Table provides the raw data on counts of injected embryos, surviving larvae and scoring of mCherry or GFP fluorescence for sparse or widespread; as well as further details on statistical tests performed in our study.

Click here to download Table S2

Table S3. Oligonucleotide primer and gRNA sequences

This Table contains CRISPR crRNA target sequences, oligonucleotide primer sequences used for RNP activity assays and F0/F1 molecular analyses and oligonucleotide primer sequences used for HDR template synthesis.

Click here to download Table S3

Development • Supplementary information

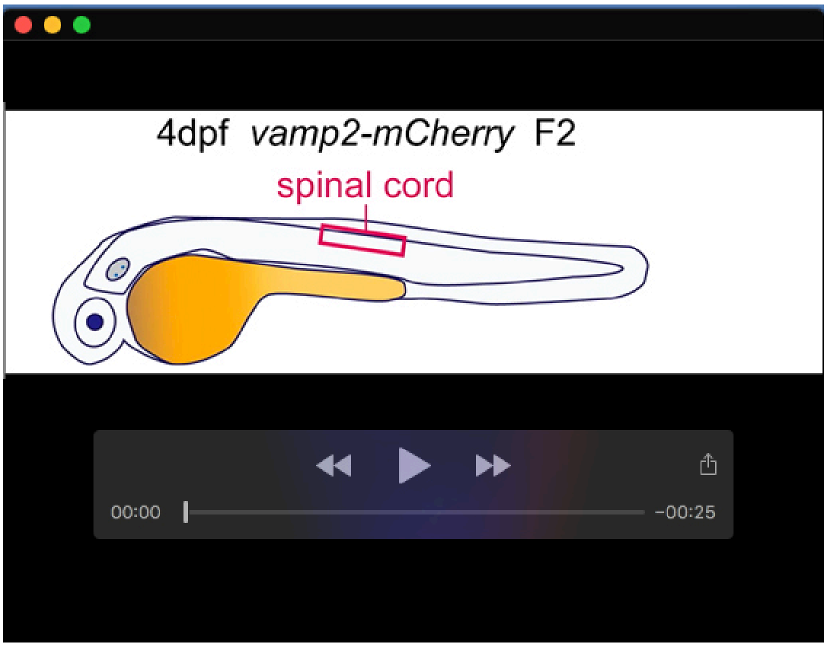

Movie 1. A z-stack through the whole depth of the spinal cord of a 4 day old *vamp2-mCherry* KI F2 embryo. Dorsal and ventral regions are indicated. Cross-section diagram indicates axon-rich areas in the peripheral tracts of the spinal cord, surrounding a neuron-rich central region. Axons show higher basal levels of Vamp2-mCherry in their membrane, compared to the neuron-rich central area, where Vamp2-mCherry is mainly punctate (i.e. vesicular).

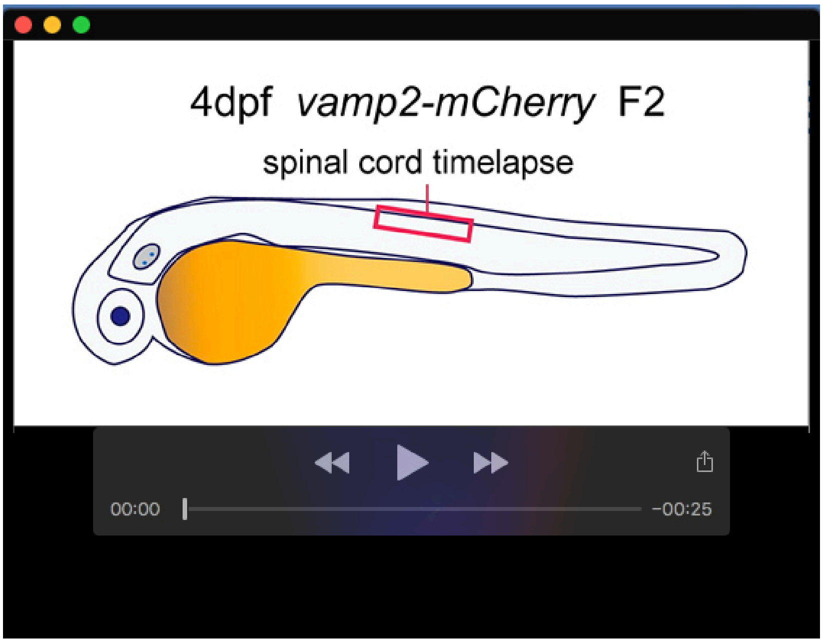

Movie 2. A ~9min timelapse of representative dorsal and ventral regions of a 4 day old *vamp2-mCherry* KI F2 embryo. This novel line will enable non-invasive analyses of the trafficking of endogenous Vamp2-bearing vesicles in vivo, without reporter overexpression.

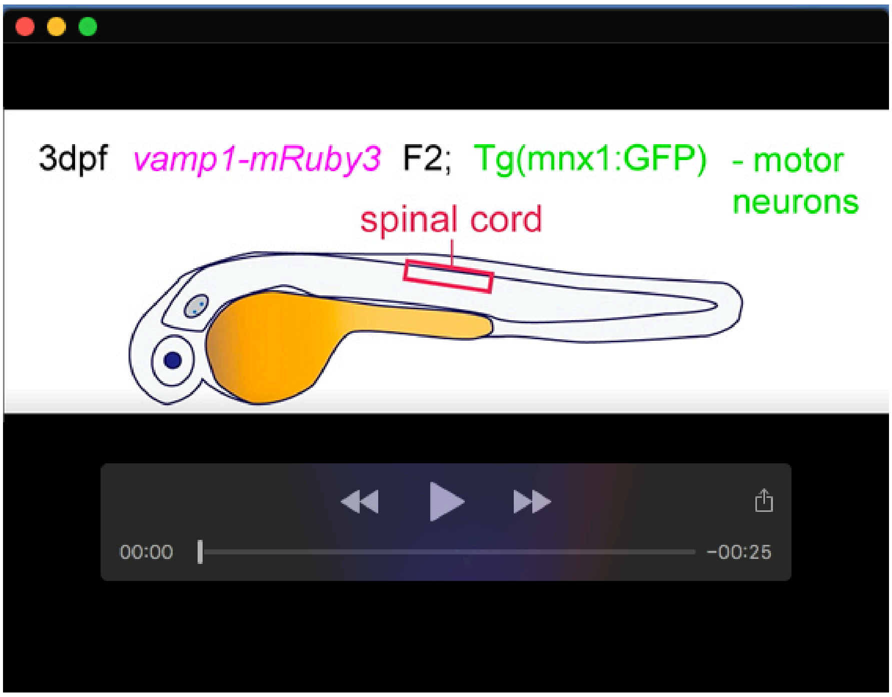

Movie 3. A z-stack through the whole depth of the spinal cord of a 3 day old *vamp1-mRuby3* KI F2 embryo that is also transgenic for motor neuron reporter Tg(mnx1:GFP). mRuby3^{+} motor axon presynaptic terminals of the neuromuscular junctions at the musculature are indicated by "NMJ".

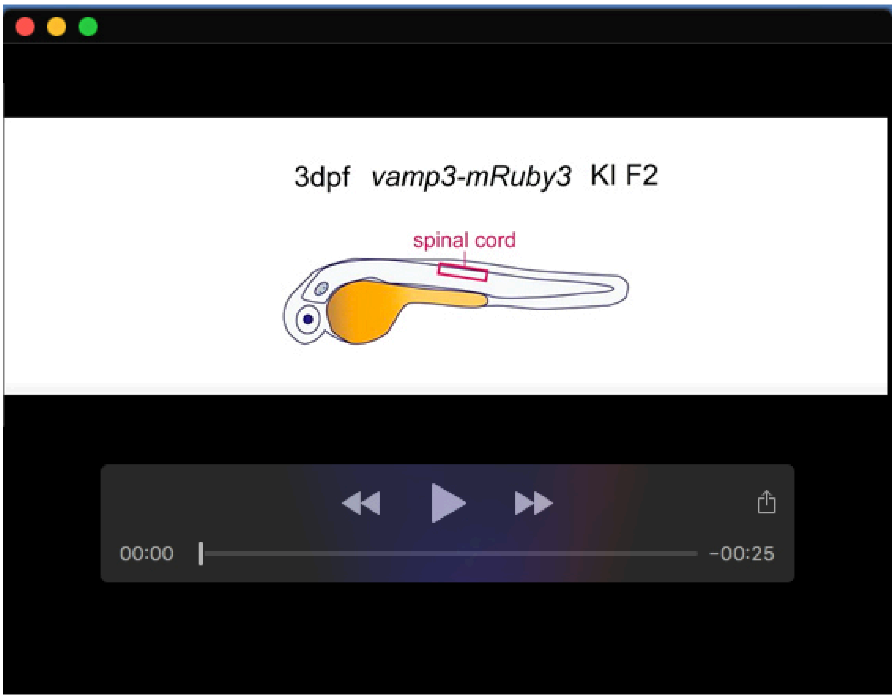

Movie 4. A z-stack through the whole depth of the spinal cord of a 3 day old *vamp3-mCherry* KI F2 embryo. Spinal cord and notochord regions indicated. Note predominantly membrane localization of fluorescent signal, in contrast to the vesicular pattern of Vamp1/2 homologues.

Supplementary Materials and Methods

Step-by-step knock-in protocol

Here, we provide a detailed protocol covering all main steps we used for in-frame reporter sequence knock-in into zebrafish genes. In this approach, we generated double-stranded DNA breaks (DSB) in target genes using the CRISPR/ *Streptomyces pyogenes* Cas9 protein system, which requires a NGG sequence for cleavage; and included a double-stranded DNA homology-directed repair (HDR) template with short homology arms to the DSB region flanking a fluorescent reporter coding sequence. For N-terminal tagging, the knock-in cassette should be integrated immediately after the endogenous start codon; for C-terminal tagging, knock-in should be integrated immediately before the stop codon. We knock-in a fluorophore reporter sequence in frame with the target gene to generate a direct fluorophore-protein fusion, or interspersed with a short 'self-cleaving' 2A peptide that enables separate cell-labelling fluorophore expression and untagged endogenous protein expression. We have been able to achieve reporter expression at 3-5 days post-fertilization in up to ~50% of the F0 injected generation, and germline transmission in up to ~67% of reporter-expressing F0 founders raised to adulthood, establishing knock-in lines after a single injection round.

A. gRNA design

a. Identify endogenous start or stop codons in the genomic sequence of target gene. Compare annotated sequences in both NCBI and Ensembl databases. Start codons can sometimes span two exons. Note that direct N-terminal tagging of a transmembrane protein may require first identifying the sequence encoding the signal peptide and targeting instead the codon immediately after the signal peptide.

b. Design forward and reverse primers (20-25bp) complementary to the genomic sequence around the target start or stop codons for PCR amplification of that region (typically, maximum of ~500bp with target start or stop in the middle). These primers will be used to identify any polymorphisms in the target genomic region in the local zebrafish stocks, and to assess gRNA activity.

c. *(optional)* Use a high-fidelity polymerase e.g. Phusion or Q5 (New England Biolabs) to amplify the target region from genomic DNA of the (wildtype) stocks used for knock-in. Electrophorese the PCR product, extract the correctly sized band and perform Sanger sequencing using the PCR primers. This will reveal any polymorphisms around the target codons, which may affect the design of the crRNA sequence as well as the homology arms. In practice, given the expediency of the knock-in protocol, this step could be performed only after the first injection round, using genomic DNA extracted from the injected larva. The knock-in mix may also be microinjected into fertilized eggs of multiple wildtype strains if they are available locally, with no significant additional effort.

d. The only design restriction for CRISPR/Cas9 is the NGG protospacer adjacent motif (PAM). The DSB site should be as close as possible to the target start or stop codon. In our knock-in experments to date the predicted DSB site has been ≤12bp away from the start or stop codon, and on either side of the start or stop codon (either coding or untranslated region). Identify an NGG sequence in either strand and the 20bp upstream of the PAM (protospacer) manually or using online design tools e.g. CHOPCHOP (https://chopchop.cbu.uib.no/). Order the corresponding synthetic crRNA from a commercial supplier (e.g. IDT DNA). If multiple crRNAs can be designed, it may be beneficial to select those highest scoring in predicted on-target activity and specificity (i.e. low off-target activity), and those in which a restriction enzyme site overlaps the predicted DSB site, to facilitate testing gRNA activity in injected zebrafish. This can also be accomplished by Sanger sequencing using primers from step *c* if no restriction enzyme site is available.

e. Resuspend crRNA and tracrRNA according to the supplier's instructions. Typically, we resuspend at 100µM in IDTE buffer (10 mM Tris, 0.1 mM EDTA), which we aliquot in single-use microtubes and store at -80°C. Work with gloves, dedicated RNA pipettes, filter tips, plasticware and nuclease-free water, and spray a RNAse decontamination solution frequently on surfaces to avoid RNAse contamination.

Development • Supplementary information

B. HDR template design

f. Identify 40bp homology arm sequences to either side of the target start or stop codons. Ideally, use the sequence identified from genomic DNA of the local stock (step *c*), to tailor homology arms to the polymorphisms in the local strains. For N-terminal tagging, use 40bp of sequence up to and including the start codon for the left homology arm, which will be part of the forward primer; and 40bp of sequence starting at the first codon after the endogenous start in the right homology arm, which will be part of the reverse primer. For C-terminal tagging, use 40bp of sequence up to and excluding the endogenous stop codon in the left homology arm, and 40bp of sequence starting at the endogenous stop codon in the right homology arm. In practice, 40bp-long homology arms have yielded sufficient knock-in, but can vary between 30-60nt.

g. To design primers for the production of the HDR template, it may be helpful to assemble the predicted PCR amplicon/knock-in cassette sequence *in silico* (e.g. we use *Ape*, a plasmid editor).
- Start with the coding sequence for the fluorescent reporter. For direct fusions to target proteins, we recommend that only truly monomeric fluorescent proteins are used e.g. GFP derivatives that include the A206K monomerizing mutation, and alternatives to mCherry – consult FPbase (fpbase.org).
- To reduce the likelihood that off-target integration results in fluorophore expression, remove the ATG start codon. Remove also the stop codon, which would stop translation at N-terminal knock-in, and is not necessary for C-terminal knock-in as the homology arm will include the endogenous stop codon.
- For direct protein tagging, we include a short linker between reporter and target, GGGGS (encoded by *ggaggcggagggtcc*) downstream or upstream of the reporter sequence for N-terminal or C-terminal knock-ins, respectively.
- Alternatively, for cellular expression tagging, include a 2A peptide sequence downstream of the reporter sequence. We use the P2A sequence (GSGATNFSLLKQAGDVEENPGP) downstream of myristoylated (membrane-tethered) fluorescent protein sequences in N-terminal knock-ins.
- Add 40bp homology arms (step *f*) to either side of the reporter sequence.
- If the gRNA target site (PAM sequence and 20bp upstream) remains intact in this predicted HDR template sequence, it is important to introduce sequence alterations to prevent continuous cleavage by persistent gRNA/Cas9 activity even after successful knock-in. A simple way to do this is to modify a nucleotide in the PAM sequence to disrupt the consensus NGG. If these changes fall within the endogenous coding sequence, be careful to ensure any codon changes are synonymous and do not alter the endogenous protein sequence.
- The primer sequences to be ordered consist of 40nt of homology arms plus ~20nt of the reporter sequence. Ensure that at least 15-20nt at the 3' end of each primer perfectly match the reporter sequence in an existing plasmid to be used as a template in the high-fidelity PCR. In practice, the exact length of homology arms and the rest of the primer can be optimized if the PCR does not yield sufficient product (e.g. extending reporter-specific sequences to ensure sufficient annealing between primers and plasmid template in the PCR).
- Order modified primers, including a 5'-end Biotin moiety and phosphorothioate bonds in the first 3-5 nucleotides. These modifications protect the linear dsDNA PCR product against concatemer formation and exonuclease degradation. We have typically ordered 100nmol scale primers with standard desalting purification (IDT DNA).

h. Resuspend oligonucleotide primers in water to 100µM concentration, and store at -20°C.

C. HDR template synthesis

i. Set up a high-fidelity PCR reaction according to supplier's recommendations to amplify the HDR template, using the modified primers and a plasmid containing the fluorescent reporter sequence. Assemble the reaction on ice and work in an RNAse-free manner as much as possible. A final

Development • Supplementary information

volume of 50µL-100µL yields sufficient product, but we have occasionally scaled this up to 200µL for low-yield PCRs. We have used both Phusion and Q5 successfully, but have found that with Phusion certain PCRs require optimization – e.g. supplementation with +1mM magnesium chloride transformed a zero-yield to high-yield reaction. With Q5, amplification has been more robust, and we have always included the High GC Enhancer.

A typical reaction set-up is:

**Component**	**Volume**
Nuclease-free water	to 50µL
5x Buffer	10µL
10mM dNTPs	1µL
10µM forward primer	2.5µL
10µM reverse primer	2.5µL
template DNA (plasmid)	(up to 10ng)
Additive e.g. DMSO	(up to 6%)
2U/µL Polymerase	0.5µL
Final volume	50µL

Typical thermocycling conditions for ≤1kbp HDR templates are:

	**Step**	**Temp.**	**Duration**
1x	Initial denaturation	98°C	30s
30x	Denaturation	98°C	10s
	Annealing	50-72°C	30s
	Extension	72°C	30s
1x	Final extension	72°C	2min

j. Run the PCR reaction in a 1% agarose gel to determine if a single, specific band was amplified.

k. Excise the band and extract the DNA using a purification kit. We use New England Biolab's Monarch Gel Extraction kit, eluting in a small volume - 6µL – of nuclease-free water.

l. Measure the concentration of the eluted product e.g. on a Nanodrop (we typically achieve 100-200ng/µL from 50-100µL PCR reactions, respectively). Store at -20°C.

D. Knock-in mix assembly and injection

m. Hybridize crRNA and tracrRNA to form sgRNA: add 1µL 100µM crRNA, 1µL 100µM tracrRNA and 3µL duplex buffer (30 mM HEPES, pH 7.5; 100 mM potassium acetate) to a PCR tube, incubate at 95°C for 5min and let cool down to room temperature gradually. This forms 20µM sgRNA.

n. Assemble the injection solution on ice:

**Component**	**Final Conc.**	**Volume**
20µM Cas9	3.2µM	0.8µL
10X Cas9 Buffer	1X	0.5µL
20µM sgRNA	4µM	1µL
HDR template	50-100ng/µL	up to 2.6µL
HDR enhancer	variable	0.1µL
H2O		(to 5µL)
Final volume		5µL

Incubate at 37°C for 10min, and then keep on ice until microinjecting. In practice, we keep unused sgRNA solution at -20°C, re-heating at 95°C and cooling down as in step *m* before re-using. In our hands, using equimolar 4 µM Cas9 and 4µM sgRNA often leads to injection needle clogging, as has

Development • Supplementary information

been described for RNP delivery of CRISPR-Cas9 reagents (Burger et al., 2016 *Development*), hence the slightly reduced Cas9 concentration.

o. Load 2-3µL of injection solution into microinjection borosilicate capillary needles pulled to a fine taper. Break the tip of the needle under a dissecting microscope using fine forceps and calibrate the injection bolus using a stage micrometer overlaid with mineral oil. Adjust injection pressure/time settings to dispense a bolus 0.1mm in diameter, which corresponds to a volume of 0.5nL. Collect freshly laid eggs, ensuring they are at the one-cell stage, and carefully inject twice into the single cell or as close as possible within the yolk. In practice, we pre-sort one-cell stage eggs to line them up for microinjection, and/or use forceps to destroy any that are already lined up and are at a stage later than one-cell when microinjecting. Typically, we inject several hundred per morning.

E. F0 screening

p. Raise injected F0 embryos at 28.5°C in Hepes-buffered E3 embryo medium. Screen embryos at the end of the injection day and at 24hpf and discard unfertilized and abnormal embryos.

q. At 2-5dpf, screen larva for fluorescent reporter expression. The exact technique will depend on which tissue reporter expression is expected. For nervous system expression, we find it convenient to mount larva on their side, which they fall into naturally at 3dpf, enabling visualization of their brain and spine. Anesthetize larva using tricaine, immerse in 1.3-1.5% low-melting point agarose and mount 2-3 larva per drop on rectangular glass coverslips, adjusting their position with fine forceps. We have used a Zeiss AxioImager equipped with a 20X objective for this step of screening. While cellular expression tagging may label whole cells, direct reporter fusions to endogenous proteins may result in the labelling of very fine structures that may be difficult to visualize with lower-magnification stereomicroscopes. For the first time running a particular knock-in experiment, we recommend this higher-magnification screening to ensure the identification of all expressing larva. Mark any positive larva in the slide. We suggest ranking positive larva according to how widespread expression is. Retrieve all positive larva from the agarose, e.g. using fine scalpels to separate the agarose drop from the larva and let recover in fresh embryo medium. With practice individual users can mount and screen hundreds of larva and retrieve positive larva from the agarose in a morning. In most of our knock-in experiments to date we have identified a proportion of larva with widespread, almost non-mosaic expression. Larva with widespread expression can be raised in separate tanks and prioritized when screening for founders with germline transmission.

r. (*optional*) it may be useful to extract genomic DNA from some injected larva to test for gRNA activity and/or for on-target integration events using a PCR assay (see section F). To do this, terminally anesthetize ~8 injected larva using tricaine, add each larva to a PCR tube and use the HOTSHOT method to extract DNA: add 30-50µL of 50mM NaOH, heat to 95°C for 10min or until the tissue has dissolved, neutralize with 1/10th of the volume (3-5µL) of 1M Tris HCl pH8, and freeze until use.

F. Validation of gRNA activity and of knock-in in F0 and F1

s. (*optional*) To test gRNA activity, perform PCR using a routine *Taq* polymerase assay and primers designed in step *b* to amplify the gRNA target region from genomic DNA from injected larva (step *r*). Typically, we set up 25µL reactions using a 2x Mastermix (Onetaq, New England Biolabs), use 1µL genomic DNA and run the PCR as follows: initial denaturation 94°C 30s; 35 cycles of 94°C 30s/50-60°C 30s/68°C 30s; final extension 68°C 2min. Half the volume is then used in a restriction digest if there is a restriction site overlapping the predicted cut site for the gRNA. A wildtype/uninjected control reaction is prepared in parallel. Undigested and digested PCR products are then electrophoresed in 1-2% agarose gels to test for gRNA activity (e.g. Fig. S1). If there is no restriction site overlapping the predicted cut site, the full PCR reactions are ran in an agarose gel, and the expected bands purified from the agarose and sent for Sanger sequencing with the PCR

Development • Supplementary information

primers. Comparison between uninjected/control chromatograms and those from injected larva can indicate whether the gRNA was active if there are significant decreases in base calling from the expected cut site onwards, indicative of indels as the cells attempt to repair the DSB using non-homologous end joining mechanisms. In practice, this step may be useful if a knock-in experiment does not yield any fluorescent larva, and may indicate the need for an alternative gRNA.

t. (*optional*) to validate the observation of reporter expression in injected F0 larva as on-target F0 knock-in, a PCR similar to step *s* may be performed using genomic DNA extracted from fluorescent larva, and primers that anneal to the reporter sequence in combination with the target genomic region (from step *b*). This assay would amplify the 5' or 3' junctions of the knock-in cassette. Amplification of the correct size band using a reporter-specific primer that is not present in the wildtype genomic sequence increases the confidence that the correct locus has been targeted. Bands of interest can be purified and sent for Sanger sequencing. Subsequent validation in F1 is essential.

u. Outcross potential F0 founders with wildtype adults and screen F1 offspring for germline transmission. Reporter expression in F1 should no longer be mosaic, but the same individual founder may produce F1s with slightly different expression patterns, resulting from separate integration events in multiple embryonic cells that give rise to germline cells with different alleles. We have observed both precise and imprecise integration from the same founder. Additionally, we have observed short 20-40bp sequence duplications of the short homology arms in untranslated regions that do not affect reporter expression. Therefore, it is important to establish subsequent F2 lines from F1s that have been individually tested for correct integration through PCR and Sanger sequencing testing of the 5'and 3' junctions, as in step *t*.

Development • Supplementary information
